# Teleost Nonapeptides, Isotocin and Vasotocin Administration Released the Milt by Abdominal Massage in Male Catfish, *Clarias magur*

**DOI:** 10.3389/fendo.2022.899463

**Published:** 2022-06-30

**Authors:** K. S. Wisdom, Irfan Ahmad Bhat, Mujahidkhan A. Pathan, Chanu T. I., Pravesh Kumar, Gireesh Babu P., Pravin Walke, Sunil Kumar Nayak, Rupam Sharma

**Affiliations:** ^1^ Division of Fish Genetics and Biotechnology, Indian Council of Agricultural Research (ICAR)-Central Institute of Fisheries Education Mumbai, Mumbai, India; ^2^ Faculty of Life and Environmental Sciences, University of Iceland, Reykjavik, Iceland; ^3^ Department of Aquaculture, ICAR-Central Institute of Fisheries Education Mumbai, Mumbai, India; ^4^ Department of Aquaculture, College of Fisheries, Dr. Rajendra Prasad Central Agricultural University, Pusa, India; ^5^ Animal Biotechnology, ICAR-National Research Centre on Meat Chengicherla, Boduppal Post Hyderabad, India; ^6^ National Center for Nanoscience and Nanotechnology, University of Mumbai, Mumbai, India

**Keywords:** stripping, milt, nanoparticles, catfish, nanotube

## Abstract

In the present work the nonapeptides i.e., isotocin and vasotocin alone or in a combination were tested in *C. magur* to evaluate their effect on stripping by abdominal massage. Also, we used chitosan-carbon nanotube nanocomposites to conjugate the nonapetides isotocin (abbreviated as COOH-SWCNTCSPeP) and isotocin and vasotocin (COOH-SWCNTCSPePs) with the aim of sustaining the effect for a longer duration. The conjugation of nonapeptides with nanocomposites was confirmed by Fourier-transform infrared spectroscopy (FTIR), Scanning electron microscopy (SEM), Transmission electron microscopy (TEM), Thermogravimetric analysis (TGA) and X-ray photoelectron spectroscopy (XPS). Two experiments were conducted to study the effect of naked (without nanoparticles) and conjugated nonapeptides on the milt release by stripping. Both the experiments consisted of eight treatments which included four naked groups two nanoconjugated groups and two controls. Both naked and nonconjugated formulations were successful in stripping the male catfish. The mRNA expression of selected reproductive genes was analysed to decipher the effect of nanopeptides at the molecular level. Nonapeptide treatment either naked or nanoconjugated, resulted in the upregulation of the transcript level of genes. Histological analysis revealed the concentration of spermatozoa was more in peptide injected groups than in the controls. The synergistic effects of nonapeptides and Ovatide had a positive impact on GSI. Thus, the present formulations were successful in stripping the male catfish to obtain the milt with significant reproductive success. Even though the naked groups perform better but the number of males required to fertilize the eggs in nanoconjuagted groups was smaller making it worth using for the delivery of nonapeptides.

## Introduction

*Clarias magur* is an Indian freshwater catfish commonly known as “Magur” (1, 2). It has a high nutritional profile and is categorized as highly-priced fish among the freshwater species. It breeds freely in wild conditions, however, in captivity, it is difficult to breed the fish even after hormonal injections. The major constraint is that the male does not ooze out milt by stripping to fertilize the eggs (3). Therefore, for induced breeding, the male magur is being sacrificed to collect milt suspension from testis (4) which may lead to a decline in the entire male population if not properly checked. There have been a lot of attempts to strip *C. magur* males using different formulations (5–9) but no significant success was achieved. So there is a prime need to modify the available hormonal formulations that have been used to stimulate the reproductive physiology in fish. Nonapeptides are the hormones of choice to be used for the purpose due to their multiple reproductive roles in fish (8, 10–12).

Nonapeptides comprise nine amino acid residues and disulfide bridges between cysteine residues at positions 1 and 6 in the peptide chain, allowing them to form a ring-like structure (13). Isotocin and Vasotocin in teleost which are homologs of oxytocin and vasopressin in mammals are well-known nonapeptides having reproductive functions. These nonapeptides are secreted from the hypothalamus and stored in the posterior pituitary (14). They act as neurotransmitters or neuromodulators in the central nervous system (CNS), and when released into the bloodstream, they act as hormones (15). In higher animals, oxytocin is known to have its specific hormonal role in promoting powerful uterine contractions during parturition (14) stimulating milk ejection during suckling in lactation, and also promoting social interaction as well as mating behaviors. While in males, it stimulates a penile erection, promotes spermiation, sperm transport and increases sperm concentration (16). Commercially available oxytocin has been used for milk ejaculation in cows (17) and sheep (18). In fish, nonapeptides are known to play a role in pair-bonding/courtship, spawning reflex and parental care (10, 19, 20).

So keeping in view the above functions, we hypothesized nonapeptide delivery could not only be helpful to induce spawning but also in oozing out milt by stripping in male magur.

Therapeutic peptides are usually unstable and often display a short half-life period in the bloodstream, so need a delivery agent that can enhance their properties (21). Here we are first time using the single-wall carbon nanotubes (SWCNT) for the delivery of nonapeptides. Carbon nanotubes (CNTs) have many advantages in terms of high surface area (22, 23) high payload of drugs inside and outside of the tubes with higher loading efficiency (24–29), good electrical and excellent thermal conductivity properties for biosensing and controlled release of drugs (30–32). CNTs are considered a promising vehicle, however, using naked SWCNT alone caused toxicity such as oxidative stress, DNA damage, apoptosis *in vivo* as well as *in vitro* (33). Therefore, due to this reason, the functionalization of CNTs with polymers is necessary to reduce the toxicity of CNTs (34).

So in the present study, we designed a nanohybrid of SWCNT and Chitosan (CS) for the delivery of nonapeptide (Teleost isotocin and vasotocin) in *C. magur*. The aim of the formulation was to check the effect on different parameters on magur including mRNA expression, hormonal profile and reproductive output.

## Materials and Methods

### Immobilization of Nonapeptides With COOH-SWCNTCS Hybrid Nanoparticles

Two teleost nonapeptides (isotocin and vasotocin supply by Allianz BioInnovation with Purity: >95% Andheri East, Mumbai-400072, India) for bony fish were used in the study. The nonapeptides were modified with a disulfide bond at C1-C6 to hold internal structural integrity and amided at N-terminal (-NH_2-_) to act as capping. The designed Nonapeptides were procured from Allianz BioInnovation, Mumbai, India. The synthesis and characterization of modified acid-functionalized single-walled carbon nanotube (COOH-SWCNTCS) hybrid are discussed in our previous study (34). The conjugation of nonapeptide with COOH-SWCNTCS hybrid was based on the methods given by Li et al. (35). Both the nonapeptides (isotocin and vasotocin) (5 mg) were completely dissolved in 10% acetic solution in two different flasks and added drop by drop into the solution containing 5ml of COOH-SWCNTCS hybrid nanoparticles under magnetic stirrer at 5000 rpm at room temperature for 3 h. Thereafter, the excess unbound nonapeptides were separated by centrifugation (12000 rpm for 30 min). Hence, the results of developed products were designated as nanoconjugated nonapeptide (isotocin only) (abbreviated as COOH-SWCNTCSPeP, represent as E) and nonapeptides (isotocin and vasotocin) (abbreviated as COOH-SWCNTCSPePs, represented as F). Similarly, naked nonapeptides are represented as A (naked isotocin only) and B (naked isotocin-vasotocin) injected after 12 h of the first injection and another group C (naked isotocin only) and D (naked isotocin-vasotocin) injected at same time points with the first injection. Both the conjugated as well as naked nonapeptides solutions were stored at 4°C.

### Entrapment Efficiency of Nonapeptide

The amount of the nonapeptides (isotocin and vasotocin) conjugated with the COOH-SWCNTCS hybrid was calculated by Lowry’s method (36). The number of free nonapeptides in the supernatant was measured at k=600nm by UV vis spectrophotometry.

### Characterization of COOH-SWCNTCSPePs

To investigate the surface morphology of COOH-SWCNTCSPeP, Field Emission Gun-Scanning Electron Microscopes (FEGSEM) (model: JSM-7600F, JEOL Ltd, Tokyo, Japan) and Field Emission Gun- Transmission Electron Microscope 300 Kv (HR-TEM 300 kV) (Model: Tecnai G2, F30, FEI, Oregon, USA) was done. To study the thermal decomposition behavior of hybrids COOH-CNTCSPePs, Thermogravimetric analysis (TGA) (Perkin Elmer, Massachusetts, USA), was performed. Fourier Transform Infra-red Spectroscopy (FTIR) (model: 3000 Hyperion Microscope with Vertex 80, Bruker, Germany) was done to study the presence of functional groups in the samples. X-ray photoelectron spectroscopy (XPS) (PHI Electronics, Chanhassen, USA, PHI 5000 Versa probe scanning ESCA microprobe using Al-Kα as the X-ray source) was performed to investigate the elemental analysis of the hybrid sample.

### Animals and Experimental Design

The present experiment was conducted at Balabhadrapuram freshwater fish farm of Kakinada center, ICAR-CIFE, Andhra Pradesh, India. Two experiments were carried out in the present study. *C. magur* males of 1+-year-old with an average weight of 127.28 ± 3.7 g were used for both the experiments while *C. magur* females of 1^+^ years old with an average weight of 89 ± 7.2 g were used for stripping eggs. The animals were harvested from the earthen ponds and acclimatized in FRP tanks with proper aeration for five days.

Experiment-1 consisted of eight groups of treatments and in each group, 6 fish were kept in duplicate. All the treatment groups received Ovatide™ as the first injection except for negative control. The reason behind Ovatide™ injection as the first injection is to regulate and stimulate the reproductive axis (GnRH). Since nonapeptides mainly function on muscle contraction and maintenance of the water channel of the reproductive organ, incorporation of GnRH (Surat, Gujarat, India) is required for induced reproduction. So in experiments 1 and 2, the treatment groups are N, P, A, B, C, D, E and F respectively. The details of treatments are presented in **Table 1**.

**Table 1:** Framework of experimental design.

	Time			
	Latency period 18 h			
	5 pm	5 pm	5 am	11 am
Treatments	1^st^ injection	2^nd^ injection*	3^rd^ injection	Stripping**
N	Negative control (PBS)	x	x	–
P	Positive control (Ovatide^TM^)	x	x	–
A	Ovatide^TM^	x	Isotocin_12pi	+
B	Ovatide^TM^	x	Isotocin_vasotocin-12pi	+
C	Ovatide^TM^	Isotocin_1	x	+
D	Ovatide^TM^	Isotocin_vasotocin_1	x	+
E	Ovatide^TM^	CNT_Isotocin_1	x	+
F	Ovatide^TM^	CNT_Isotocin_Vasotocin_1	x	+

*1^st^ and 2^nd^ injection-Single time * Second injection- after 12 h.

(X) No injection.

(-) Not response on stripping.

(+) Response on stripping.

**Stripping: after 18 h.

Inducing hormones “Ovatide™” was administrated uniformly in all the treatments except the negative control. Since Ovatide™ contains GnRH which constitutes 10 amino acids (decapeptides), whereas, nonapeptides consist of 9 amino acids, Therefore, the present study focus on the synergistic effects of both aiming that it might increase the performance, particularly for the *C. magur* under the captive environment. Therefore, for the present study, the injection dose was decided based on the concentration of GnRH content (standardized) in the Ovatide™ i.e., 20µg/ml. Ovatide™ of 0.2ml/200gm body weight was given to all males in our experiments. This dose was five times more increased in the Balabhadrapuram environment than the recommended dose. Thus, we have decided an injection dose of 8µg/200gm of body weight of *C. magur* (based on the Ovatide™ GnRH concentration). In positive control, only Ovatide™ was injected whereas in negative control the animals were injected with PBS with the same dose. After intramuscular injection, the fish were released into the tank, and stripping was performed after 18h. Serum and gonads were collected from fish in the first experiment after 18h for genes expression studies and hormonal assay

Experiment-2 was conducted as a repetition of Experiment-1 keeping the same group of treatments to confirm the reproduction (in consecutive years) results. The second experiment was used for stripping purposes and to study reproductive performance.

### Ganado Somatic Index

GSI was conducted to study the development in the gonads due to the effects of nonapeptides. For this study, equal numbers (6 duplicate) of male brooders were sacrificed from different groups of treatments, and gonads were collected. The gonadosomatic Index (GSI) was estimated (37) by,


$$\text{GSI}=\frac{\text{Gonad weight}}{\text{Total body weight}}\times 100$$

### Hormone Levels in Serum

The effect of naked and nanoconjugated nonapeptides on gonadal hormones, such as, Maturation Inducing hormones (MIH), Follicular Stimulation hormones (FSH), Luteinizing hormones (LH), and Testosterone (T) in the serum samples was quantified by enzyme-linked immunosorbent assay (ELISA) kit (Bioinnovation, Mumbai, India) followed to the guidelines provided by the manufacturer. The absorbance was measured in Biotek microplate reader (Winooski, VT. USA) at a wavelength of 450 nm. The analysis was done based on Rathor et al. (38) and Rather et al. (39).

### Gene Expression by Real-Time PCR

The mRNA expression of transcription factors involved in the steroidogenesis pathway and hormonal genes like FSHβ and LHβ was carried out in testes collected from different treatment groups at different time points post-injection. The transcriptional factors include StAR (Steroidogenic acute regulatory protein), CYP11a1a (cytochrome P450 family 11), Cyp17a1a (cytochrome P450 family 17), 3β-hsd (3β-hydroxysteroid dehydrogenase) and17β-hsd (17β-hydroxysteroid dehydrogenase). qRT-PCR was performed in Roche Light Cycler 480 thermocycler using 2× Syber Green mix (Thermo Fisher Scientific, USA). The primers for qRT-PCR were first serially diluted to check the efficiency of primers and the tissues were run with a β-actin housekeeping gene. The reactions were set in 25 µl of total volume using 2 µl of diluted cDNA, 1 µl of each forward and reverse primers 12.5 µl of Syber green mix and nuclease-free water to make up the volume. The PCR cycle consisted of an initial denaturation step at 94°C for 30s, followed by an amplification step (40 cycles of 95°C for 5 s/60°C for 15 s/72°C for 20 s), a single melting curve step of 95°C for 5 s/65°C for 15s/95°C and finally a cooling step of 40°C for 30s. The fold change in mRNA expression data was analyzed using the 2−ΔΔCt method (40). The primer used for the mRNA expression study is shown in (**Supplementary Table 1**).

### Tissue Collection and Preparation for Histology

The tissues for the preparation of histological slides were dissected aseptically for both experiment 1 and experiment 2 after treatments and fixed in neutral buffer formalin (NBF). Histology of gonads was performed as per Luna (41) and Rather et al. (39). The slides were photographed using an FSX100 Olympus, Tokyo, Japan inverted microscope.

### Statistical Analysis

Data were statistically analyzed by using SPSS-16.0 Version software expressed as mean ± SEM in one-way analysis of variance (ANOVA) followed by Duncan’s multiple range tests. P <.05 was considered statistically significant. For analysis of stripping success, fertilization rate and hatching rate, Software SAS (online free version) has been used.

## Results

### FTIR Results of COOH-CNTCSPePs

**Figure 1:**
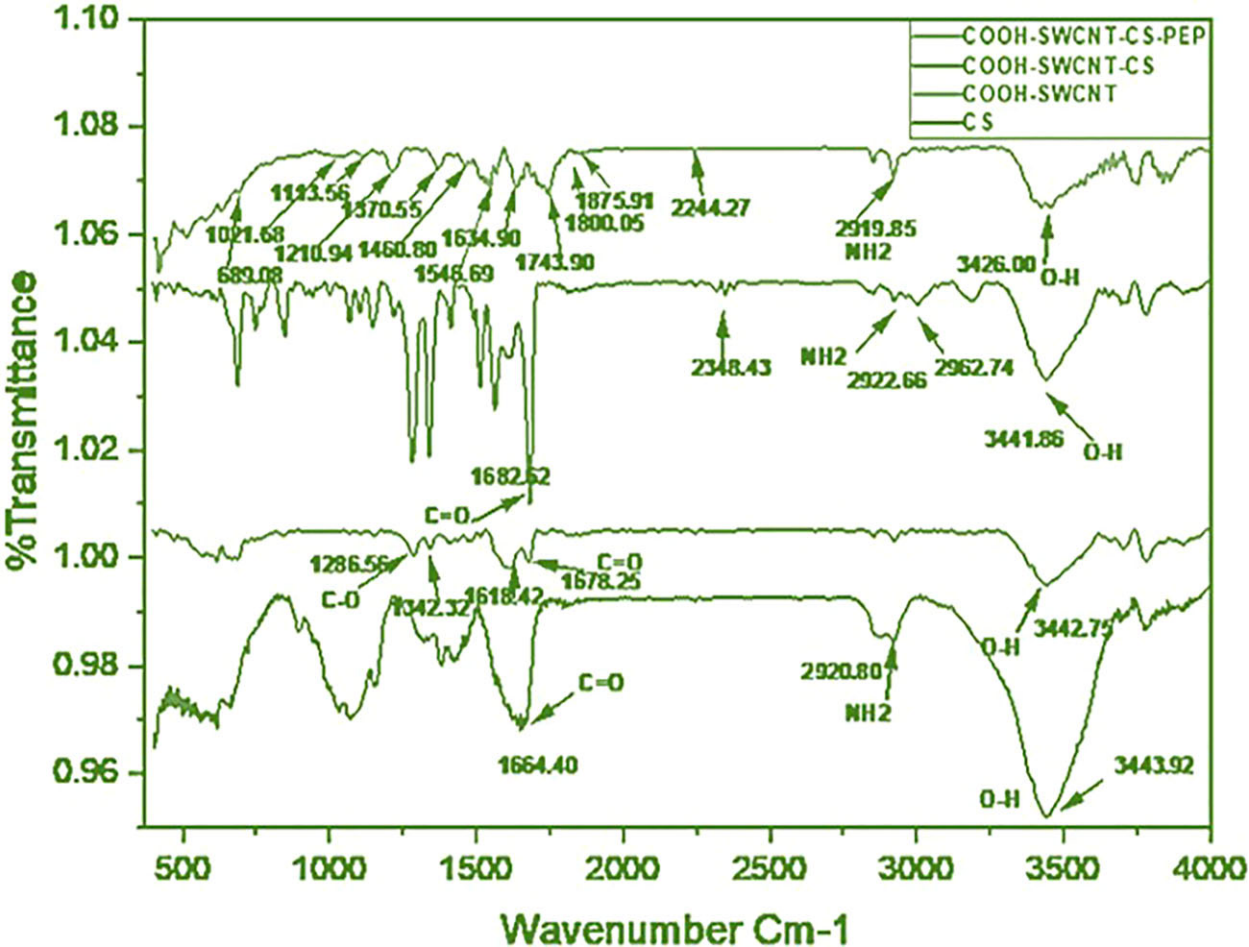
FTIR of CS (chitosan), COOH-SWCNT (single-walled carbon nanotubes), COOH-SWCNTCS (single-walled carbon nanotubes with chitosan), COOH-SWCNTCSPePs (Chitosan-carbon nanotubes with nonapeptides).

The FTIR spectra showed a peak at 2000 and 2700 cm^-1^ referring to the backbone of SWCNT. Absorption bands in the range 3,000–3,500 cm^−1^ (3,440) are related to the presence of the hydroxyl group. A peak at 3442.75 cm^-1^and 1678.25 cm^−1^ found in COOH-SWCNTCSPePs indicated the incorporation of hydroxyl(O-H) and carboxylic group (COOH), respectively. Similarly, peaks at 1682.62 cm^-1^, 2922.66 cm^-1^ and 3441.86 cm^-1^ were found which attributes OH, NH_2_ and C=O incorporated from the CS (chitosan) with slight shifting (35). In, COOH-CNTCSPePs, peaks detected at 1210.94 cm^-1^ are assigned for C-N stretching, 1113.56 cm^-1^ for C-O stretching, 689.09-1021.68cm^-1^ for N-H stretching of the nonapeptides and peak at 1564 cm^-1^ attributed the presence of secondary amide of nonapeptides on the COOH-SWCNT-CS. All this shifting and overlapping of the peaks confirmed that the nonapeptides are successfully conjugated over the COOH-SWCNTCS. As shown in **Figure 1**.

### SEM Image of COOH-SWCNT and COOH-CNTCSPePs

**Figure 2:**
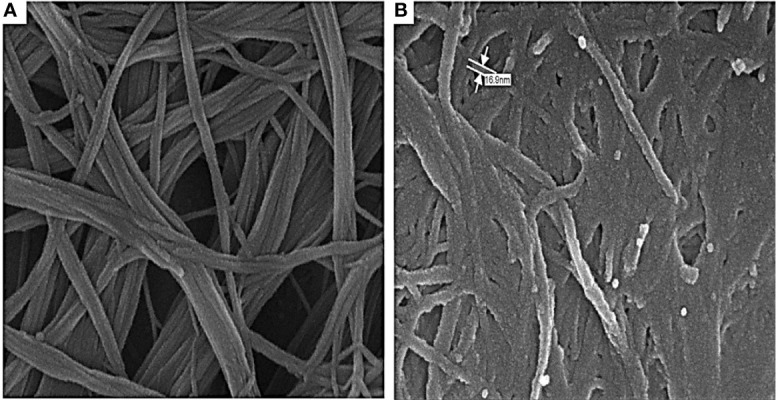
SEM results of **(A)** COOH-SWCNTs (single-walled carbon nanotubes) and **(B)** COOH-SWCNTCSPEPs (Chitosan-carbon nanotubes with nonapeptides).

The morphology of COOH-SWCNT and COOH-CNTCSPePs was analyzed by SEM. COOH-SWCNT appeared in micron length sizes with a diameter of 16 nm (**Figure 2**). On the other hand, in COOH-CNTCSPePs distinctive texture appearance was observed because of CS and nonapeptides grafted in the nanohybrid (**Figure 2**).

### TEM Image of COOH-SWCNT and COOH-CNTCSPePs

**Figure 3:**
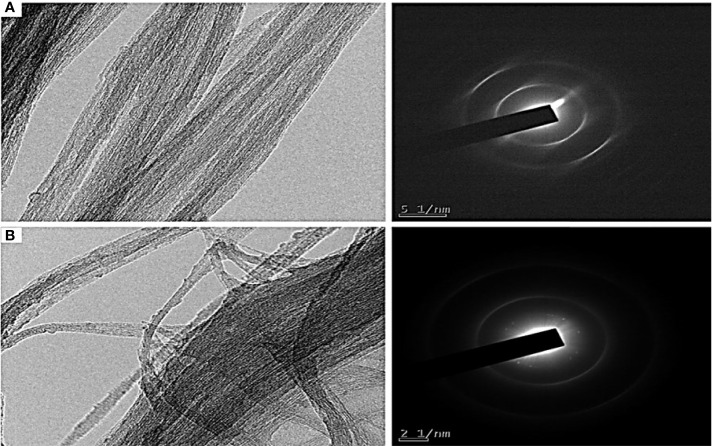
TEM results of **(A)** COOH-SWCNT (single-walled carbon nanotubes) and **(B)** COOH-SWCNTCSPePs (single-walled carbon nanotubes with nonapeptides).

Further, the morphology of COOH-SWCNT and COOH-CNTCSPePs was examined by TEM. It showed a rough surface and black dots on the wall in COOH-CNTCSPePs, compared to the COOH-SWCNT; this distinctive appearance shows CS and nonapeptides were incorporated. On the other hand, for COOH-SWCNT complete smooth clean single wall tubes were visible (**Figure 3**).

**Figure 4:**
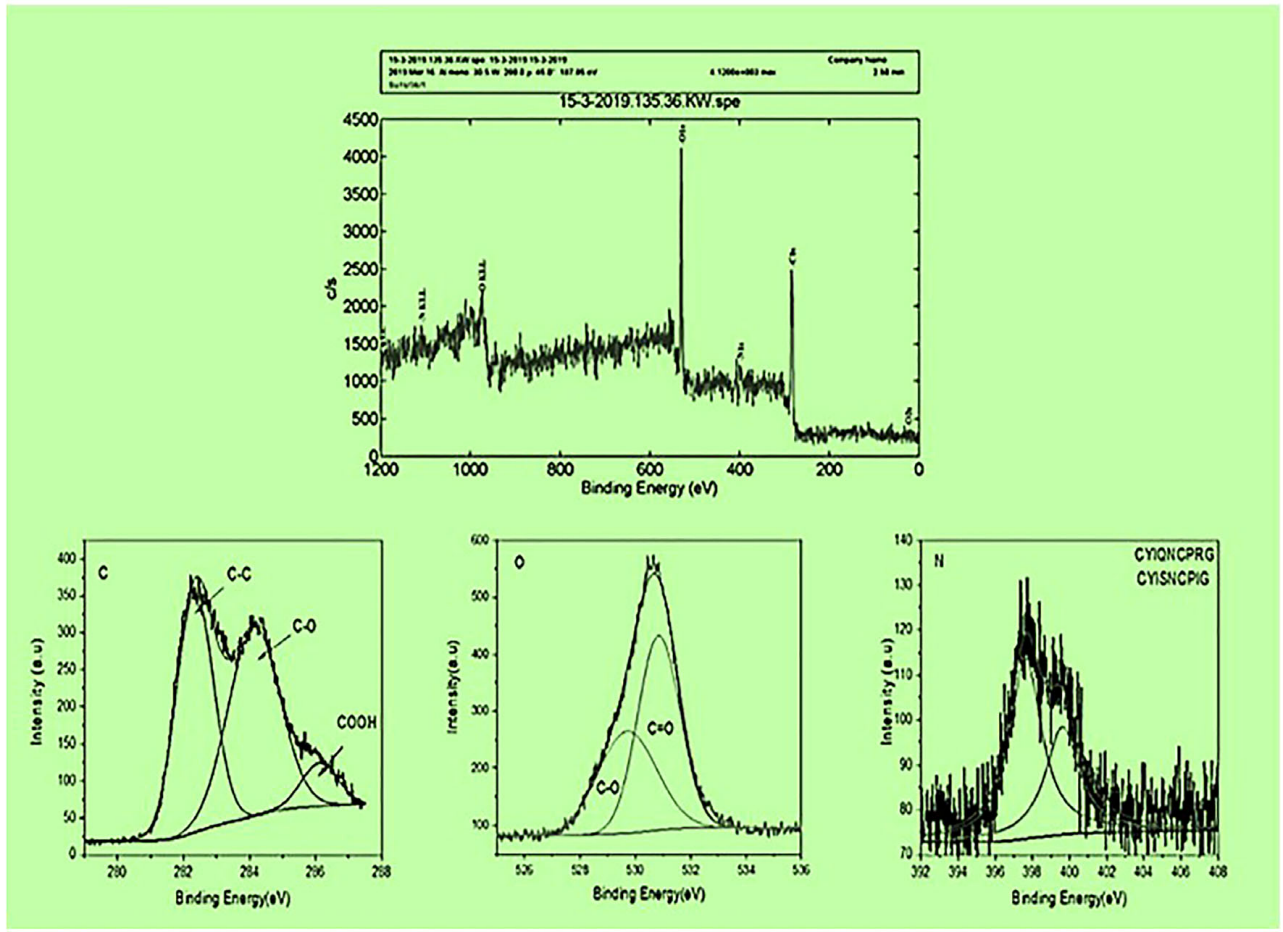
XPS results of COOH-SWCNTCSPePs (single-walled carbon nanotubes with chitosan and nonapeptides).

To ensure the surface elemental composition of the CS-SWCNT-COOH nonapeptides conjugated sample, XPS analysis was conducted. The spectrum resulting from the sample showed the presence of carbon, oxygen and nitrogen in the COOH-CNTCSPePs sample. Hydrogen was not detected since XPS is not sensitive to Helium or hydrogen. The C1s (state of the binding energy of carbon) core level peak positions of the carbon atoms are approximately 284.5-290.5 eV. The peak position for oxygen is centered at around 526.6-532.6 eV and the N1s core level peak positions of the nitrogen are approximately 390-408 eV. Due to mixed acid H_2_SO_4_:HNO_3_ oxidation XPS of C1s reveals the formation of carboxylic and carbonyl on the COOH-CNTCSPePs. No residue spectrum of catalysts iron or cobalt was detected in the sample. The peak positions in COOH-CNTCSPePs are at 530.3, 531.5, 533.2, and 535.6 eV, which correspond to the binding energies of C−O, C=O or lattice oxygen, hydroxyl group and chemisorbed oxygen or water, respectively. As shown in XPS spectra of COOH-CNTCSPePs (**Figure 4** and **Supplementary Table 2**), the characteristic binding energy of 284.4 eV, 285.4 eV, and 286.9 eV, was attributed to C-C, C-O, COOH, respectively. As expected, the SWCNT network exhibits a Carbon peak (∼284.5 eV) and an Oxygen peak (∼532 eV) arising from physically adsorbed oxygen and oxygen groups on the defect sizes. NH_2_ (400.23–400. 44 eV).

### TGA Results of COOH-CNTCSPePs

**Figure 5:**
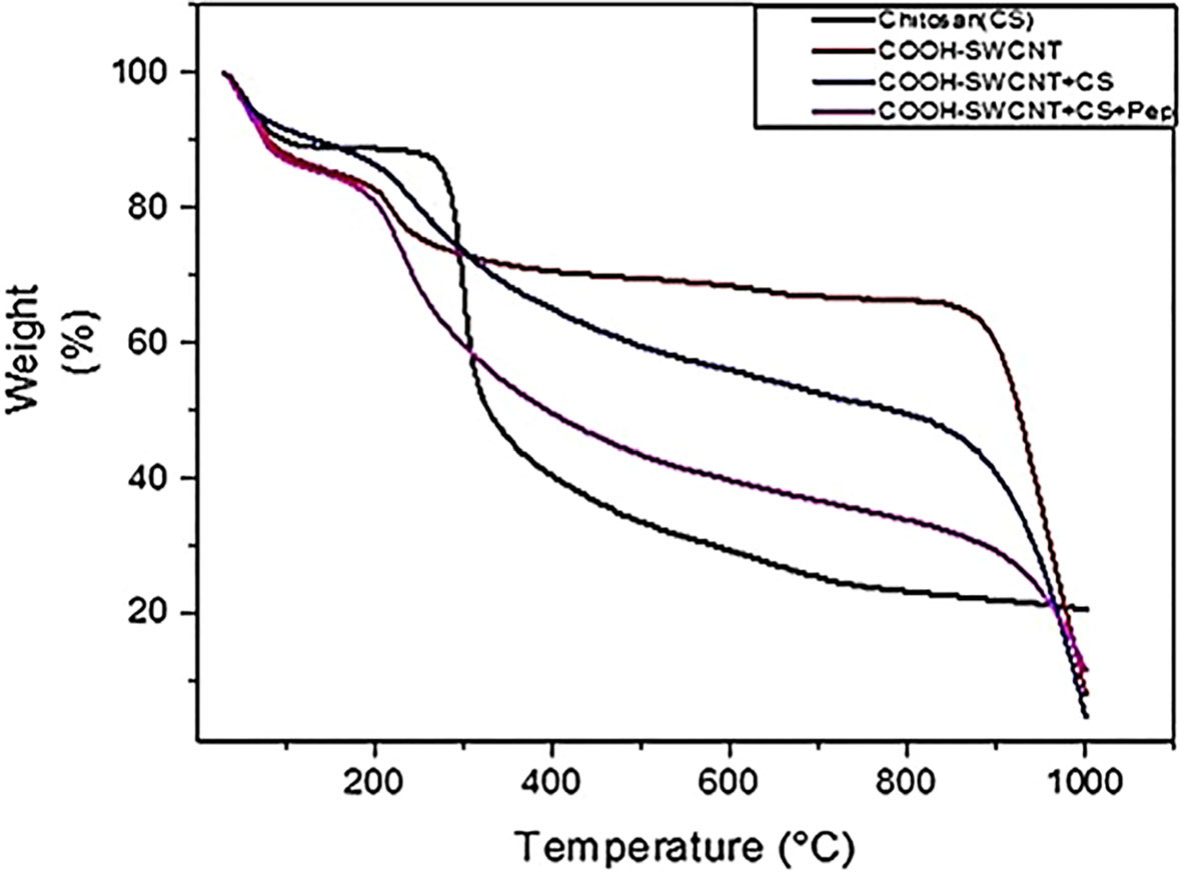
TGA results of CS (chitosan), COOH-SWCNT (single-walled carbon nanotubes), COOH-SWCNTCS (single-walled carbon nanotubes with chitosan), COOH-SWCNTCSPePs (Chitosan-carbon nanotubes with nonapeptides).

The TGA for COOH-CNTCSPePs weight loss was observed at 100°C, 200-400°C and finally at 800-1000°C. Weight loss at 100°C could be due to thermal breakdown of nonapeptide and weight loss at 200°C was due to the breaking down of amine group and glycosidic bond of CS. There is a weight loss that occurred at 250°C and constant weight loss up to 800°C and then a sharp decrease at 850°C-1000°C. This is due to the effect of acid treatment which resulted in more defects and anchored-COOH group in SWCNT. This shows the characteristic of carbon nanotubes. The TGA for CS, COOH-SWCNT, COOH-SWCNTCS are presented in our previous report (34). As shown in **Figure 5**.

### Entrapment Efficiency

The entrapment efficiency of nonapeptides on SWCNT-COOHCS was calculated to be 80%.

### Breeding

In the negative control (N) and positive control (P), stripping trials were conducted but brooders failed to respond. On the other hand, stripping of injected male brooders for each treatment was performed, resulting in a successful releasing of milt from the papilla as shown in (**Supplementary Figure 1A**).

### Collection of *C. magur* Milt

The oozed-out milt was collected in the Petri plate containing 2-5ml of 0.8 physiological saline with the help of a pipette. The same collection protocol was followed in all the treatment groups. The pictorial presentation of the milt collection is shown in (**Supplementary Figure 2B**).

### The Milt/Sperm of *C. magur* Under 100x Microscope Image

To confirm the presence of sperm in the milt, a few diluted drops from the Petri plates were taken on the slides. The slides were air-dried at room temperature for 10-15 mins And finally, stained with 10% Giemsa solution. The prepared slides were dried properly at room temperature under the fan and washed 2-3 times with DDW to remove the excess stains. The images of sperm were visible having doted head and tail (**Supplementary Figure 3**).

### Stripping Success

The stripping success is a binary trait with two-level responses ‘Yes’ and ‘No’. The logistic procedure was invoked with stripping success as the response variable. The factors viz., bodyweight of male (grams), batch (batch 1 and 2), type of particle (Nano form and normal), hormone frequency (one, two and three), booster (yes or no) and treatment (six Nona peptide formulations and 1-Ovatide control and another PBS control) were tested individually for their effect on stripping success adopting following model.


$${\text{Y}}_{\text{ij}}=\text{μ}+{\text{factor}}_{\text{i}}$$

Y_ij_ – is response to stripping success “Yes” or “No”

Factor_i_-effect of body weight of male/batch/type of particle/hormone frequency/booster/treatment

The bodyweight of males had no significant effect on stripping success. The confidence interval for odds ratio passes through one indicating no significant difference as shown in (**Supplementary Figure 4**). Similarly, treatment shows no significant effect on stripping success. The confidence interval for the odds ratio passes through one indicating no significant difference as given in (**Supplementary Figure 5**). The batch had no significant effect on stripping success. The confidence interval for the odds ratio passes through one shows no significant difference as given in (**Supplementary Figure 6**). Similarly, for booster dose had no significant effect on stripping success and confidence interval for odds ratio passes through one indicating no significant difference as given in (**Supplementary Figure 7**). The effect of hormone frequency had no significant effect on stripping success. The confidence interval for the odds ratio passes through one indicating no significant difference as given in (**Supplementary Figure 8**). A similar pattern was observed for factor particle type and total hormone which has no significant effect on stripping success. The confidence interval for the odds ratio passes through one indicating no significant difference as given in (**Supplementary Figures 9**, **10**), respectively. The joint test for all the factors is shown in **Table 2**.

**Table 2:** ANOVA for factors, body weight of male, treatment, batch, booster, hormone frequency, type of particle, total hormone (Joint test).

Factor	DF	Wald Chi-Square
Body Weight of male	1	1.08^NS^
Treatment	5	3.1516 ^NS^
Batch	1	0.5300 ^NS^
Booster	1	2.38 ^NS^
Hormone frequency	1	2.38^NS^
Type of particle	1	0.9072^NS^
Total hormone	1	0.2558^NS^

-indicates significant difference, NS-indicate non-significant difference.

Further, the LSMEANS and ANOVA were also performed and the effects of the batch, treatment and replicates within treatment on the percentage of stripping success were estimated by invoking PROC GLM in SAS. The percentage value of stripping success was transformed to logistic values before analysis. Tukey’s test was used for comparison between groups. The following model was adopted,

Y_ijkl_= μ+ batch_i_+ treatments_j_+ replicate (Treatments)_jk_ +e_ijk_

Y_ijk-_ Response to stripping success

μ- Mean

Batch_i_-effect of batch where i = 1 and 2

Treatment_j_-effect of treatment where j=1 to 8

A- Isotocin_12pi

B- Isotocin_vasotocin-12pi

C- Isotocin_1

D- Isotocin_vasotocin_1

E- CNT_Isotocin_1

F- CNT_Isotocin_Vasotocin_1

N- NC_1_PBS

P- PC_1_Ovatide™

Replicate (treatments)_k_ - effect of replicate where k= 1 and 2

e_ijk_- error

The least-square means for batch (**Table 3**) and treatments are shown in **Table 4**. and ANOVA table is given in **Table 5**. The treatments had a significant effect on the percentage of stripping success. The treatment groups except for the Ovatide**™** and PBS group yielded stripping success. However, there was no significant difference in stripping success percentage between the different forms and combinations of nonapeptide treatment. The effect of batch and replicate (treatment) were found to be non-significant. The estimate mentioned in the text is logistic values.

**Table 3:** Least Squares Means of stripping percentage for batch 1and 2.

Batch	Stripping LSMEAN
1	0.61**^a^** ±0.598
2	0.60**^a^** ±0.005

The same superscript suggest non-significant difference at P>0.05.

**Table 4:** Least Squares Means for stripping percentage across the treatments.

Treatment	Stripping LSMEAN
CNT_Isotocin_1	0.62**^b^** ± 0.010
CNT_Isotocin_Vasotocin_1	0.63**^b^** ±0.010
Isotocin_1	0.64**^b^** ±0.010
Isotocin_vasotocin_1	0.62**^b^** ±0.010
Isotocin_12pi	0.67**^b^** ±0.010
Isotocin_vasotocin-12pi	0.65**^b^** ±0.010
NC_1_PBS	0.50**^a^** ±0.010
PC_1_Ovatide^TM^	0.50**^a^** ±0.010

The different superscript suggest significant difference at P>0.05.

**Table 5:** ANOVA for effect of batch, treatment and replicate (treatment).

Source	DF	Mean Square
Batch	1	0.001
Treatment	7	0.017^*^
Replicate(Treatment)	8	0.0006
Error	15	0.0004
R^2^ (in %)	95	

*-indicate significance ( p < 0.01).

Pairwise comparison using the Tukey grouping indicates no significant difference across the batches (**Supplementary Figure 11**).

Pairwise comparison using the Tukey test indicates no significant difference across the treatments as shown in (**Supplementary Figure 12**). However, no difference is observed in the treatment involving nonapeptide formulations (**Supplementary Figure 12**).

### Fertilization and Hatching

Instantly, the collected milt was mixed with the quality eggs taken 1:1 (male: female) ratio without a delay within 1-2 min for successful fertilization and to prevent the milt/sperm quality from deterioration (**Supplementary Figure 13**).

After well mixed with milt, the eggs were attached to the *Eichhornia* plant roots (sterilized with formalin) shown in (**Supplementary Figure 14**). The attached eggs were transferred to each plastic tank and kept for incubation and were constantly observed. Fertilized eggs start hatching after 18-19h of incubation.

### Percentage of Fertilization Rate and Hatching Rate

After milt was collected, we evaluated the fertilization rate and hatching rate. The LS means for fertilization rate is given in **Table 6**. in which fertilization is formed into two groups B, C and A treatment under one group and rest D, F and E under another group. However, treatments B, C and A show significant differences from treatments D, F and E groups.

**Table 6:** Least squares means of fertilization rate for various treatments.

Treatment	LSMEAN
Isotocin_12pi	0.68**^a^** ±0.003
Isotocin_vasotocin-12pi	0.68^a^±0.003
Isotocin_1	0.68^a^±0.003
Isotocin_vasotocin_1	0.67^b^±0.003
CNT_Isotocin_1	0.66^b^±0.003
CNT_Isotocin_Vasotocin_1	0.66^b^±0.003

Superscript “a” and “b” suggest significant difference at P>0.05.

Pairwise comparison using the Tukey grouping also indicates two groups B, C and A treatments in one group and D, F and E in another group as shown in (**Supplementary Figure 15**).

Similarly, the LS means for hatching rate is given in (**Supplementary Table 3**) where treatment A is significantly different from the rest of the treatments. However, treatments B, C, D, E and F show the non-significant difference from each other as shown in (**Supplementary Figure 16**).

From the ANOVA test we found out that the means square for fertilization rate is found significant (P<0.001). However, for the hatching rate, the effects of treatments are found non-significant. The ANOVA table for fertilization and hatching rate is given in (**Supplementary Tables 4** and **5**).

### Counting of Hatchling

After the hatching of eggs was completed, the unfertilized whitest eggs and *Eichhornia* roots were removed from the tub. The hatchlings were allowed to remain undisturbed. After the yolk absorption was completed, counting of hatchlings was performed carefully. The hatchling of each group of treatments is shown in (**Supplementary Figure 17**).

### Gonadosomatic Index

The GSI was significantly different in all the treatment groups compared to the control with the highest in treatments A and E followed by the rest treatment groups. A similar trend in the GSI was found in experiment 2 as shown in (**Supplementary Figure 18**).

### Experiment-1: Histopathological Analysis of Testis

**Figure 6:**
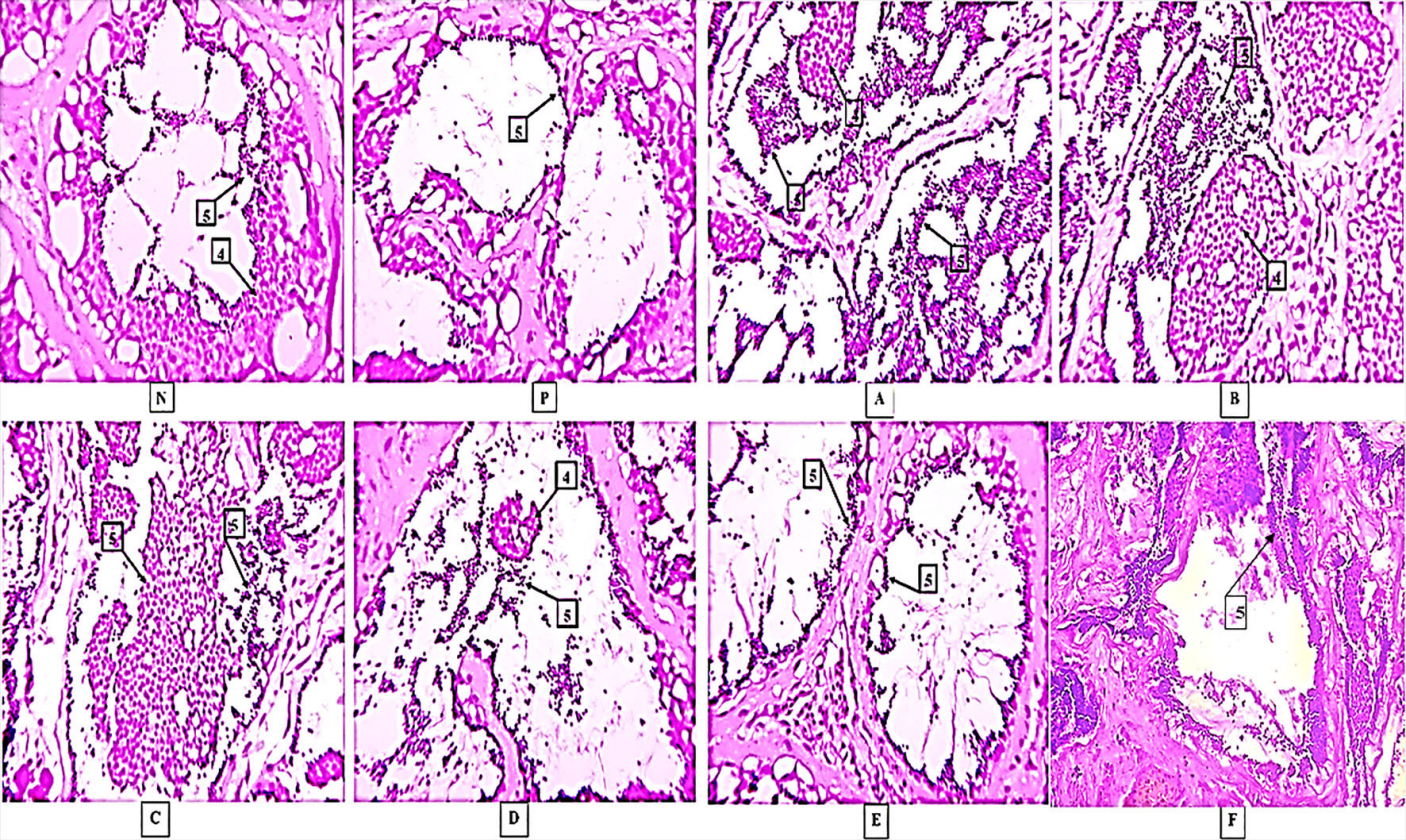
Histological results in different treatments. Here, [N] Negative control and [P] Positive control 4-Spermatids, 5-Spermatozoa, **(A–F)** are treatments. The photographs were taken at a magnification of 40x.

The mature spermatozoa were detected in almost all the treatments including control groups. The lobules were occupied fully with spermatozoa in all groups. However, more dense spermatids and spermatozoa were observed in group A and group B treatments, whereas, in both the control groups a few spermatids and spermatozoa were detected. The histopathological analysis of testes collected after experiment-1 is shown in **Figure 6**.

### Experiment-2: Histopathological Analysis of Testis

**Figure 7:**
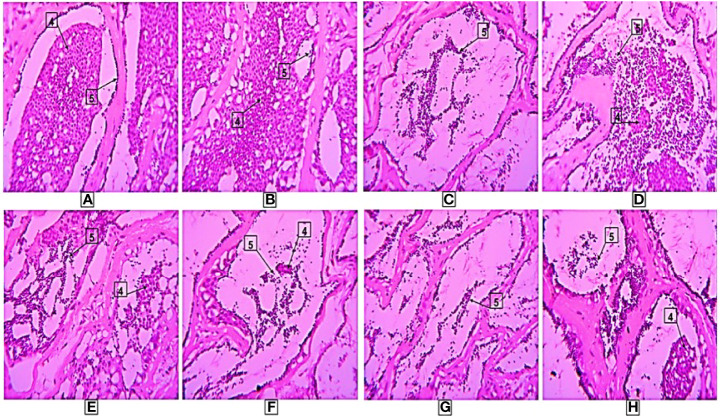
Histological results in different treatments. Here, 4-Spermatids, 5-Spermatozoa, **(A–F)** are treatments. The photographs were taken at a magnification of 40x.

Similarly, in experiment-2, the mature spermatozoa were detected in all treatment groups including the controls. The presence of numerous amounts of spermatids and spermatozoa were detected in the large lumens of testicular lobules in treatments A, B and C while in D and E groups comparatively fewer spermatids and spermatozoa with large lumens were found. On the other hand, in nano formulated group F, a large number of spermatids and spermatozoa were observed. The histopathological observations for different treatments in experiment-2 are shown in **Figure 7**.

### Expression Analysis of Different Reproductive Genes in *C. magur*

The mRNA expression analysis of reproductive genes involved in the process of steroidogenesis and gonadal development was carried out in testes collected at different time points of post-injection.

#### 3βhsd

All the treatments showed an increasing trend in the mRNA expression level compared to the controls with the highest level in A and B groups followed by C and E groups of treatments. While mRNA levels in treatments D and F showed a similar trend.

#### 17βhsd

The mRNA expression of 17βhsd was significantly increased in all the treatment groups compared to the control with the highest in A, followed by E while in B, C, D, and E groups the expression trend was similar.

#### Cyp17a1a

The significantly high expression was found in group A followed by groups B and E. mRNA expression was the same in C, D and F with respect to controls but was significantly lower than in other treatment groups.

#### Cyp11a1a

Significantly higher expression was recorded in group A. Cyp11a1a mRNA level was significantly low in treatment F compared to the remaining treatment groups but was more than in the control groups.

#### StAR Gene

In all the treatment groups, the StAR mRNA level was significantly higher than in the control groups.

#### LH

The LH mRNA levels were significantly higher in all the treatments compared to the controls but in group A, a high surge in the expression was recorded followed by B and C a similar trend was observed in D, E and F treatments.

#### FSH

**Figure 8:**
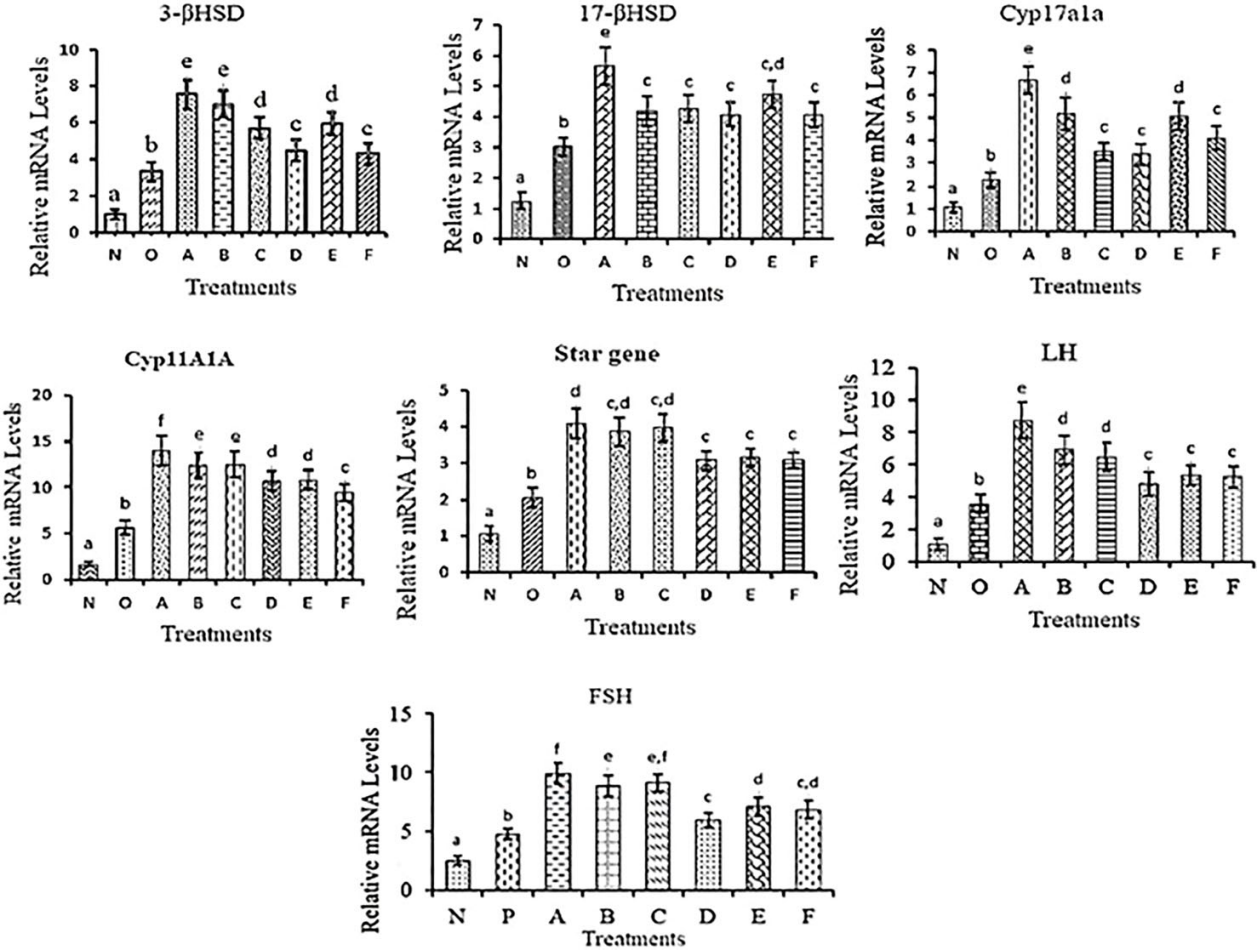
mRNA expression levels of different reproductive genes in testes collected at different time points in different treatment groups. The different letters used indicates significant differences and superscripts indicate non-significant differences in mRNA expression level. Data were expressed as mean ± S.E.M.

The mRNA expression of FSH was significantly increased in all the treatment groups compared to the control with the highest in A, followed by C and then B while in E and F groups shows a similar level of mRNA expression. However, in treatment D, a low level of mRNA expression was observed compared to other groups of treatments. The mRNA expression profile is presented in **Figure 8**.

### Hormonal Profile Studies of Injected *C. magur*

The effect of nonapeptides on reproduction was studied by analyzing the changes in the hormonal profile in the serum. For that, five different hormones like follicle-stimulating hormone (FSH), luteinizing hormone (LH), maturation inducing hormones (MIH), and testosterone (T) were analyzed in the serum collected from different treatments.

#### LH

LH level in blood serum was increased in all the groups with the highest in groups A and C. While B, D, E and F exhibited the same hormonal level but in all the treatments the serum LH level was significantly different from control groups.

#### FSH

The FSH level in blood serum was increased in groups A and E and was significantly higher than in the rest of the treatments.

#### MIH

The highest hormonal peak was found in group A followed by B, C and E, whereas, the hormonal level in groups D and F was similar. However, the MIH level in all the treatments was significantly different from the control group.

#### Testosterone

In all groups, an increased testosterone level was found compared to the control groups. Testosterone level was found highest in treatments A, B and C. In D, E and F groups same level of the hormonal surge was detected.

**Figure 9:**
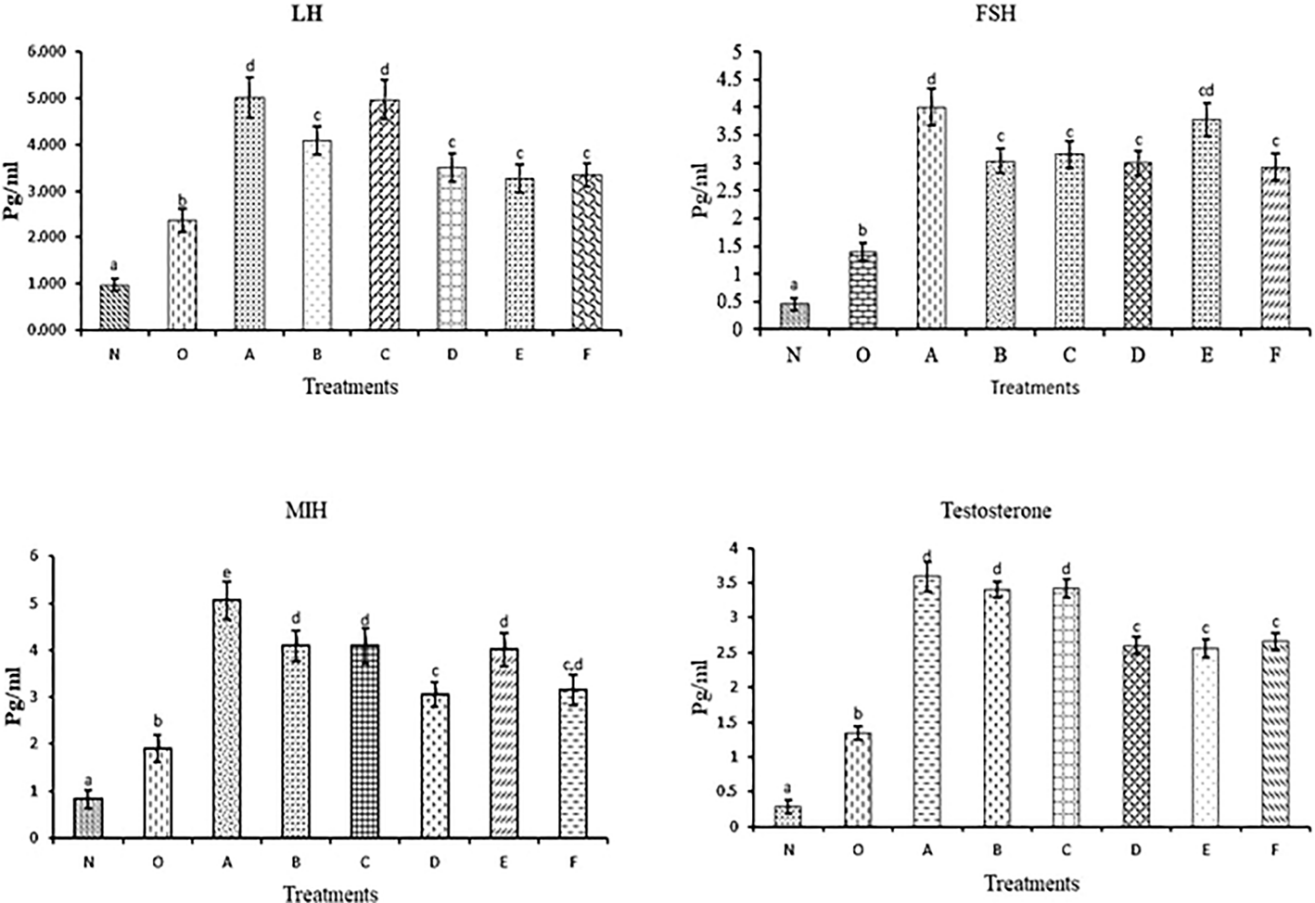
Hormonal levels in blood serum collected at different time points in a different group of treatments. The different letters used indicates significant differences and superscripts indicate the non-significant difference in hormonal levels. Data were expressed as mean ± S.E.M.

The graph of the hormonal level of different treatment groups is shown in **Figure 9**.

## Discussion

Several attempts were attempted to strip *C. magur* for milt, using different approaches. In the present study, the nonapeptides were used to release milt in *C. magur* by simple abdominal massage to avoid their sacrifice. Comparative experiments were carried out in this study using naked nonapeptides either alone or with nanocarrier (COOH-SWCNT-CS) to observe the effects on reproductive performance. However, recently some workers have successfully fertilized eggs voluntarily by using mammal-based nonapeptide oxytocin in *C. magur* (42), but, till date, this is the first time success reports in *C. magur* reproduction using teleost based nonapeptides. Our findings of this study, will help farmers to breed the male species successfully without sacrificing.

In this study single wall, CNT (SWCNT) was synthesized of diameter 20nm and length of 50µm by Chemical Vapour Deposition (CVD) (Antsprosys, India) using methane, as a source of carbon (43). The synthesized SWCNT was purified with HCl by refluxing at 80°C for 12h to remove the impurities of the catalyst. The purified SWCNT were refluxed in mixed acids (Con.H_2_SO_4_ + HNO_3_, 75:25 vol.%) at 80°C for 24h (44) to achieve carboxylation (COOH). The carboxylated SWCNT was then lyophilized and stored in a desiccator for future use. The synthesis of COOH-SWCNTCS was an in situ-based ionotropic gelation process (35, 45). Noncovalent grafting of COOH-SWCNT surface with CS reduces its toxicity when tested in fish (34). To characterize the manufactured nanohybrid, different techniques were employed. FTIR is one of the vibrant tools for detecting their functional groups. The spectra of COOH-SWCNTCSPePs have multiple common peaks, including the C–O stretch at 1113.56 cm^-1^, the C=C ring stretch at 1634.90 cm^-1^, C–H stretches at 2850 cm^-1^, the O–H stretch at 3200 cm^-1^, and the N–H stretch at 3320 cm^-1^ indicate the functional group of nonapeptides (46). FTIR results of CS, COOH-SWCNT, COOH-SWCNTCS are well explained in our previous paper (34).

The morphology and size of COOH-SWCNT and COOH-SWCNTCSPePs were characterized by SEM and TEM. In naked COOH-SWCNT, the image was observed with very smooth surfaces graphene sheet structure of SWCNT having a diameter of about 16nm, with entangled and twisted. On the other hand, in COOH-SWCNTCSPePs rough surface appears with an enlarged diameter measuring 16.9nm. This SEM image indicated that the COOH-SWCNTCSPePs nanohybrid has been developed successfully. Similar results have been reported by Yang et al. (47). In addition, TEM image results provide essential information about the incorporations of nonapeptides on COOH-SWCNTCS carriers (48, 49). To draw a complete conclusion of nanoparticle characterization, XPS analysis was done to investigate the elemental composition of nanocomposite nanohybrid. From the results of the XPS spectrum, the peak at ~392-408eV present in COOH-SWCNTCSPePs nanocomposite spectra attributes the amino group (nitrogen) of chitosan as well as the nitrogen group (nitrogen) in nonapeptides (48). At the same time, the intensity of (oxygen) O1s ∼526-536 eV peak present in COOH-SWCNTCSPePs spectra significantly increased which indicates the addition of hydroxyl group from chitosan. Strong acid oxidized SWCNT, resulted in physical defects to adsorb more oxygen groups. The peak at∼280-288 eV shown in the spectrum generally is assigned for C1s backbond of carbon of SWCNT. Similar results on XPS spectra have been reported in Wei et al. (49).

Nonapeptides play important roles in shaping as well as connecting strong social bonds (50) in three-spined stickleback and mating pairs courtship (51), in male goldfish (52). In mammals, it helps in the ejaculation of male reproductive fluids. Similarly, in fishes, it is believed to play a similar role in terms of the reproduction paradigm. Popesku et al. (53, 54), conducted a study in which sexually regressed female goldfish were treated with nonapeptide (isotocin), and it increased the LH levels. Likewise, in the present study, we found the instant hormonal surge in A and B treatments which triggered the release of inducing hormones for reproduction in *C. magur*. However, many researchers have reported that nonadelivery of hormones, drugs, etc, upregulated the reproductive hormones as well as increased the spermatozoa in fishes (5–7, 38). Similarly, in the present study nanoconjugated nonapeptides and naked nonapeptides were developed and delivered in *C. magur.* The reproductive parameters such as stripping milt, fertilization rate, hatching rate, GSI and reproductive hormonal profile such as FSH, LH, MIH, and testosterone were analysed. Our findings indicated that nonapeptides either alone or with nanoparticles surged the hormonal levels which stimulate the milt release in a short period.

Nanoconjugated nonapeptides sustained the hormones up to 16h post-injection. Similar results were reported in the previous studies (6, 7, 39). In other groups, the hormonal level was decreased with time which may be due to the short half-life of nonapeptides in the bloodstream (55). This phenomenon of increased hormonal levels in the COOH-SWCNTCS groups could be due to the slow release of nonapeptides.

In the present study, the mRNA expression of different reproductive genes was studied. The results of mRNA expression for different treatment groups draw a distinct conclusion between the nanoconjugated group and the naked group. The mRNA expression was increased in all nonapeptide injected groups mainly in groups A and B. These results clearly show that the effect of nonapeptides immediately triggers the steroidogenic pathway and thus increases hormonal production. However, in the case of treatments C and D, the mRNA expression levels were lower compared to A and B which probably maybe because of the short shelf-life of nonapeptides, since the exposure duration of these groups was comparatively longer. On the other hand, in nanoconjugated treatments, the mRNA levels were maintained almost up to 16h post-injection. This phenomenon indicated that nanoencapsulation slowly and constantly releases the drug and maintains the effect for a longer duration (6, 39, 44, 56–60). Thus, nanoformulated nonapeptides positively influence the mRNA expression level of different reproductive genes.

Mammalian nonapeptide (oxytocin) promotes spermiation and sperm transport in mice and also increases the concentration of sperm released (61). Likewise, in this study, a similar effect could be the reason behind the successful oozing out of milt on the stripping of injected male *C. magur*. In this study, we conducted two 2 experiments to analyze the stripping performance. In experiment-1, several strippable males in groups A and B were found more in strippable males while in groups C and D, the number was low. On the other hand, in the case of nanoformulated groups, a moderate number of males could be stripped, however, the fertilization rate was high. Similarly, in experiment-2, treatments A and B recorded a higher percentage of striped males than the remaining other groups. The stripped males in experiment-2 were comparatively lower than in experiment-1. This could be due to the unfavorable environmental conditions that prevailed during the breeding season, like shorter monsoon periods, late arrival of monsoon, high-temperature fluctuation, uneven heat, etc.

The significant fertilization rate, hatching rate and percentage of larvae survival were recorded in all the treatment groups with the highest percentage in treatments A and B. The effect of nonapeptides on reproductive output in fish has not been studied so far. In mammals nonapeptide oxytocin, has been shown to increase the contractility in the epididymis and to modulate the steroidogenesis (62). Oxytocin at a concentration of 10 IU/ml, was found to significantly increase the percentage of motile spermatozoa and sperm velocity than the saline controls. Oxytocin influences steroidogenesis in a Leydig cell (63, 64) and regulates the male sexual behavior leading to penile erection and ejaculation (65). Depletion of testicular oxytocin by the destruction of the Leydig cells can be associated with a decrease in spontaneous seminiferous tubule contractility which can be restored by the administration of exogenous oxytocin (66). The success of milt release through stripping achieved in the present study could be due to the effect of nonapeptides, which are considered necessary for the proper passage of sperm.

In both the experiments, the highest GSI was recorded in groups A, B and E compared to the other treatment groups. The significant increase of GSI may be due to the synergistic effect of nonapeptides and Ovatide on the reproductive physiology of catfish. Similar results have been reported in the previous studies also (67–69) indicating that nonapeptides have played an important role in reproductive parameters.

The histological analysis of testis was done to confirm the changes at the cellular level due to naked and nanoconjuagted nonapeptides. The spermatozoa and spermatids were present in the injected groups. In control groups, spermatozoa were observed in a lesser number. In both the experiments, lumens and lobules of testis in groups A and B were fully occupied by the huge number of spermatids and spermatozoa. Similar, results were recorded in the nanoconjugated groups. The results are supported by the fact that nonapeptides helped in advancing testicular development and spermatogenesis (11). The nonapeptides influence the androgen levels which in turn help in the testicular development in mice (61). Nonapeptides trigger contractions of eggs cell in fish (70). In male fish, it might have a role in the contraction of the testis which triggers the ejaculation of sperms/milt while stripping.

## Conclusion

We successfully tested the effect of nonpeptides, delivered either alone or with nanoparticles designed nontoxic naked nonapeptide on induced breeding of *C. magur*. The treatments were successful in releasing the milt from male magur by simple abdominal massaging without sacrificing the male species at recommended dosages. Even though the results of naked groups and nanoconjugated groups vary non-significantly but the number of males used during fertilization is low in the latter group making the nanoformulation worth using.

## Data Availability Statement

The original contributions presented in the study are included in the article/**Supplementary Material**. Further inquiries can be directed to the corresponding author.

## Ethics Statement

The animal study was reviewed and approved by ICAR-CIFE, Division of Fish Genetics and Biotechnology, ICAR- Central Institute of Fisheries Education Mumbai-400061, India.

## Author Contributions

KW: Original draft, Methodology, Software, Validation, Visualization, Writing - original draft. IB: Formal analysis, Visualization, Methodology, Writing - original draft, Writing - review and editing. CT: Investigation, Resources, Methodology. MP: Methodology, Investigation. PK: Formal analysis. GB: Methodology, Data curation. SK: Investigation. PW: Software, Resources. RS: Conceptualization, Methodology, Validation, Supervision, Project administration. All authors contributed to the article and approved the submitted version.

## Funding

ICAR-Central Institute of Fisheries Education, Mumbai, and NFST-Ministry of Tribal Affairs, India

## Conflict of Interest

The authors declare that the research was conducted in the absence of any commercial or financial relationships that could be construed as a potential conflict of interest.

## Publisher’s Note

All claims expressed in this article are solely those of the authors and do not necessarily represent those of their affiliated organizations, or those of the publisher, the editors and the reviewers. Any product that may be evaluated in this article, or claim that may be made by its manufacturer, is not guaranteed or endorsed by the publisher.
