# Supplementary material for: Teleost Nonapeptides, Isotocin and Vasotocin Administration Released the Milt by Abdominal Massage in Male Catfish, Clarias magur
Source: Front Endocrinol (Lausanne). 2022 Jun 30;13:899463. doi: 10.3389/fendo.2022.899463 (PMC9280678; doi:10.3389/fendo.2022.899463)
Supplement: Supplementary file 1 [file DataSheet_1.docx]

Table.1. List of primers used for qRT-PCR

| Primer name | Sequence 5’- 3' | Tm | Product length |
| --- | --- | --- | --- |
| Star-qRT-F | CGAGGAACATGACGGGTT TGAGG | **57** | **111** |
| Star-qrt-R | TCCTGCGGCGGATGTTTCT GA |  |  |
| 17βhsd-qRT-F | TCGTCCTCATCAGCCGTAC TC | **60** | **110** |
| 17βhsd-qRT-R | CCGATCCAAAGTCTGCAG AGA |  |  |
| 3βhsd-qRT-F | TGAGGGAGCCACAGACAA GAG | **60** | **123** |
| 3βhsd-qRT-R | GCCAAAGCCAAGTGGTAC CA |  |  |
| CYP17a-qRT-F | GCTCCAGCACTTTCCAAAT GAAG | **62** | **119** |
| CYP17a1-qRT-R | ACGGAGTAGAGCATCCAA CAGGTC |  |  |
| Cyp11a1-qRT-F | AGCTGATGGCTGGAGGTG TAG | **67** | **125** |
| Cyp11a1-qRT-R | CAGTGCGAGCTGCAATGA CT |  |  |
| LH-F | CCTGGCGTTTCAAACCAGCATCTG | **67** | **175** |
| LH-R | GCGACAGGATATGTGACATGGGGGATC |  |  |
| FSH-F | TGTGGGAGCTGCGTCACCATCAAC | **67** | **175** |
| FSH-R | GGTGAAGGACGAGTCAACCCCCG |  |  |
| β-actin-qRT-F | GCACCCCGTCCTGCTTACT GAG | **55** | **250** |
| β-actin-qRT -R | CCAGACGGAGGATGGCAT GG |  |  |

Tab: 2. XPS analysis results of COOH-CNTCsPePs

| **N1s** | Energy Binding/ eV  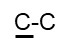  /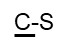 | Energy Binding/ eV  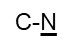 | Energy Binding/ eV  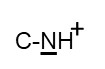 | Energy Binding/ eV  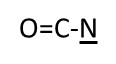/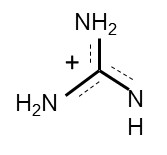 | Amino acid / peptide | Chemical formula |
| --- | --- | --- | --- | --- | --- | --- |
|  | 285 eV |  | 401eV | 402 eV | Cysteine | C_3_H_7_NO_2_S |
|  |  |  | 401eV |  | Tyrosine | C_9_H_11_NO_3_ |
|  |  |  | 401eV |  | Glutamine | C_5_H_10_N_2_O_3_ |
|  |  |  | 401eV |  | Asparagine | C_4_H_8_N_2_O_3_ |
|  |  |  | 401eV |  | Proline | C_5_H_9_NO_2_ |
|  |  | 399 eV |  | 402 eV | Arginine | C_6_H_14_N_4_O_2_ |
|  |  |  | 401eV |  | Glycine | C_2_H_5_NO_2_ |
|  |  |  |  | 402 eV | Serine | C_3_H_7_NO_3_ |

| **C1s** | C-C/C=C | C-O/>C=O | COOH |
| --- | --- | --- | --- |
|  | 285.11–285.5 eV | 286.21–287.53 eV | 289–291.6 eV |

| **O1s** | **COO^­-^** | **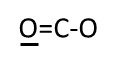** |
| --- | --- | --- |
|  | 531.3 | 532.3 |

| Tab: 3.  Least Squares Means (Hatching rate) | |
| --- | --- |
| Treatment | FR2 LSMEAN |
| Isotocin_12pi | 0.68**^b^**±0.002 |
| Isotocin_vasotocin-12pi | 0.67**^ab^**±0.003 |
| Isotocin_1 | 0.67**^ab^**±0.003 |
| Isotocin_vasotocin_1 | 0.67**^ab^**±0.003 |
| CNT_Isotocin_1 | 0.67**^ab^**±0.003 |
| CNT_Isotocin_Vasotocin_1 | 0.67**^a^**±0.003 |

| Tab: 5.  ANOVA (Hatching rate) | | |
| --- | --- | --- |
| Source | DF | Mean Square |
| Treatment | 5 | 0.0002**^NS^** |
| Error | 77 | 0.00001 |
| R^2^ (in %) | 12.3 |  |
| ^NS^-indicate non- significance ( p < 0.01) | | |

| Tab: 4.  ANOVA (Fertilization rate) | | |
| --- | --- | --- |
| Source | DF | Mean Square |
| Treatment | 5 | 0.001**^*^** |
| Error | 77 | 0.0001 |
| R^2^ (in %) | 39 |  |
| *-indicate significance ( p < 0.01) | | |
