# Supplementary material for: Teleost Nonapeptides, Isotocin and Vasotocin Administration Released the Milt by Abdominal Massage in Male Catfish, Clarias magur
Source: Front Endocrinol (Lausanne). 2022 Jun 30;13:899463. doi: 10.3389/fendo.2022.899463 (PMC9280678; doi:10.3389/fendo.2022.899463)
Supplement: Supplementary file 2 [file DataSheet_2.docx]

Supplementary file

**
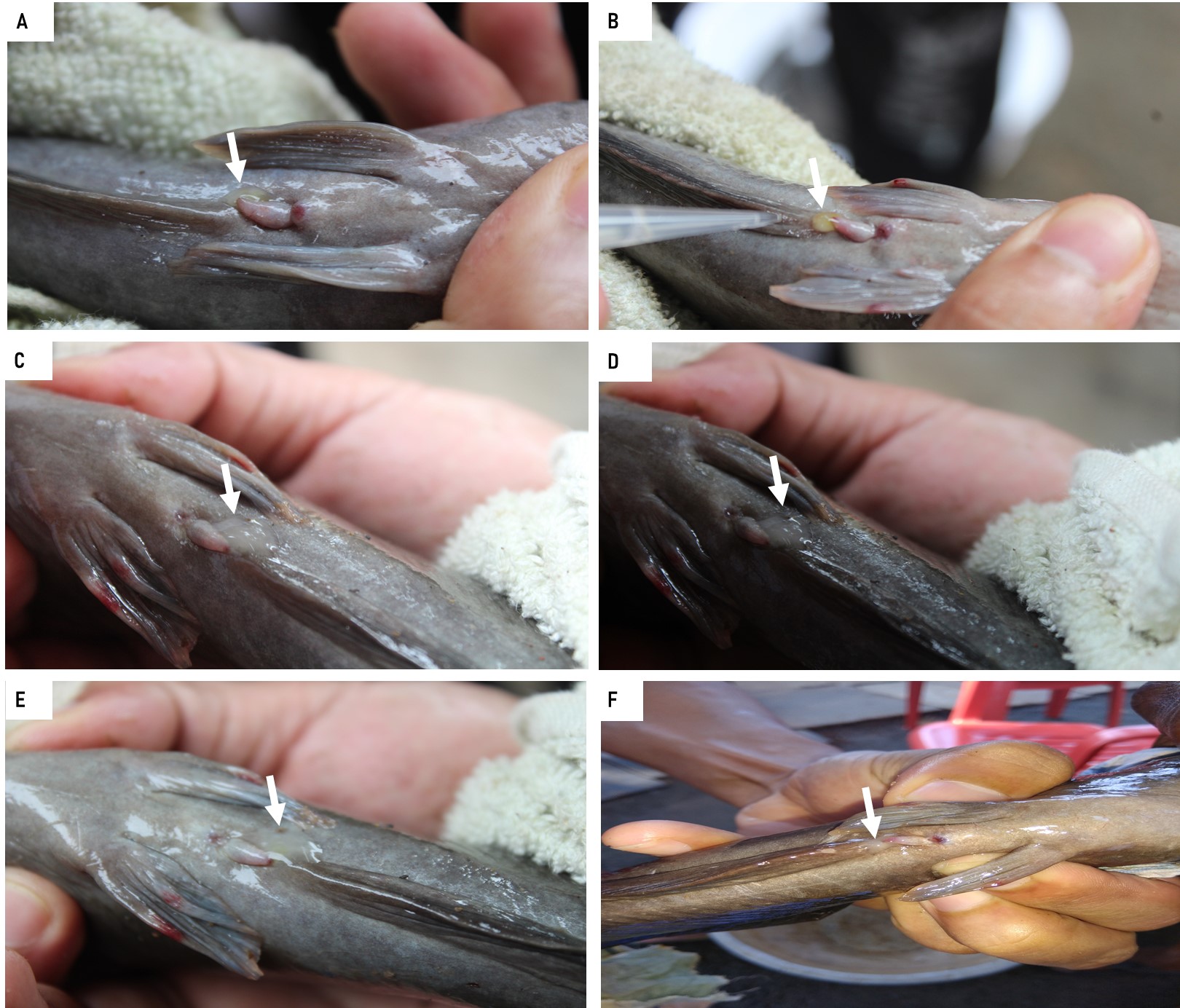
**

**
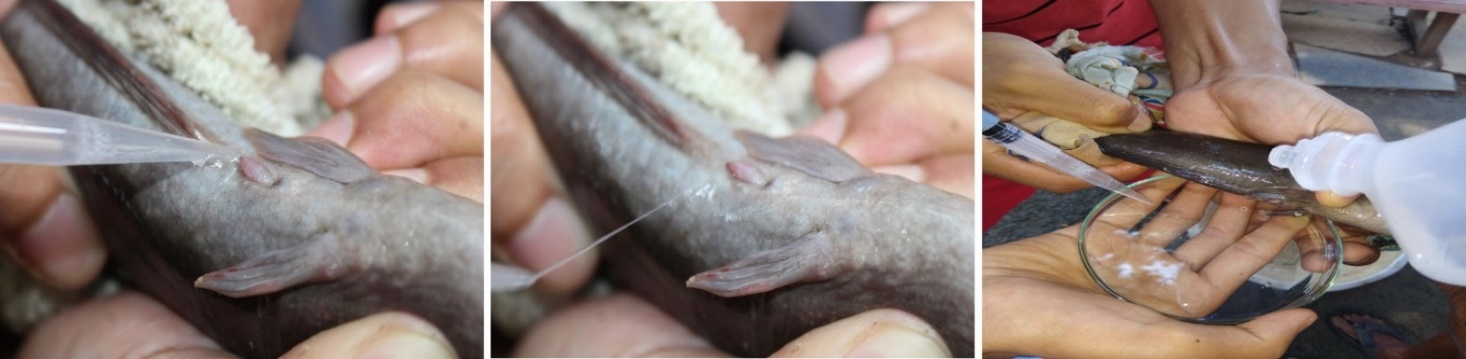
Fig.1. a. stripping of milt**

**Fig.2. b. Collection of *C. magur* milt**

**
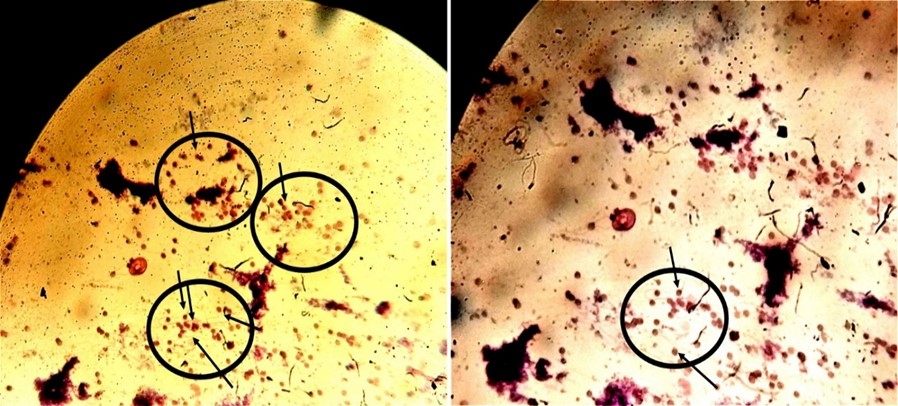
**

**Fig.3. Sperm detected in stripped milt under microscope. Magnification. 100x**


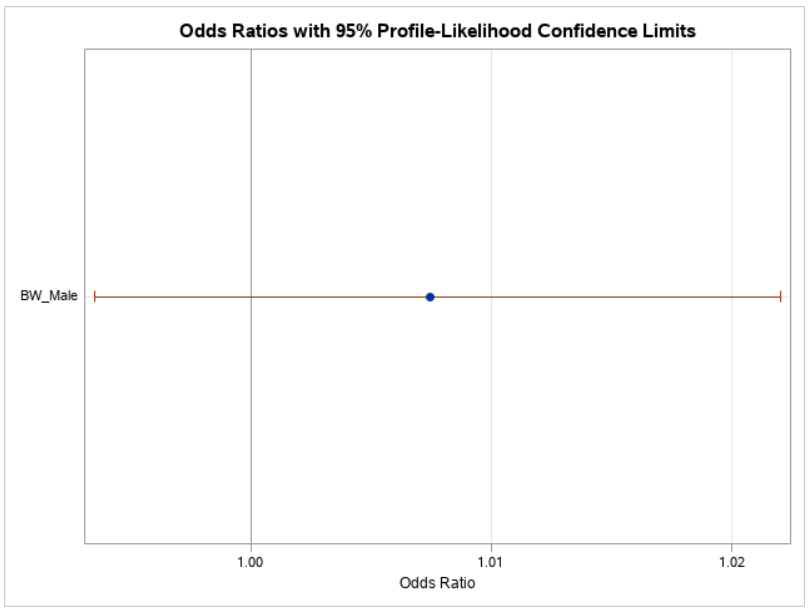


**Fig.4. Odds ratio, confidence interval for body weight**


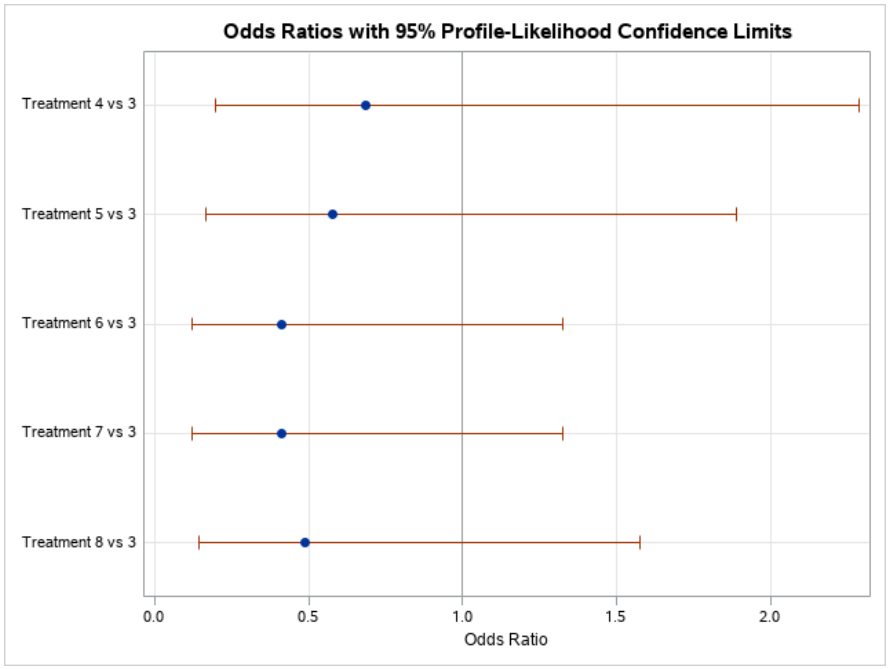


**Fig.5. Odds ratio, confidence interval for treatment**


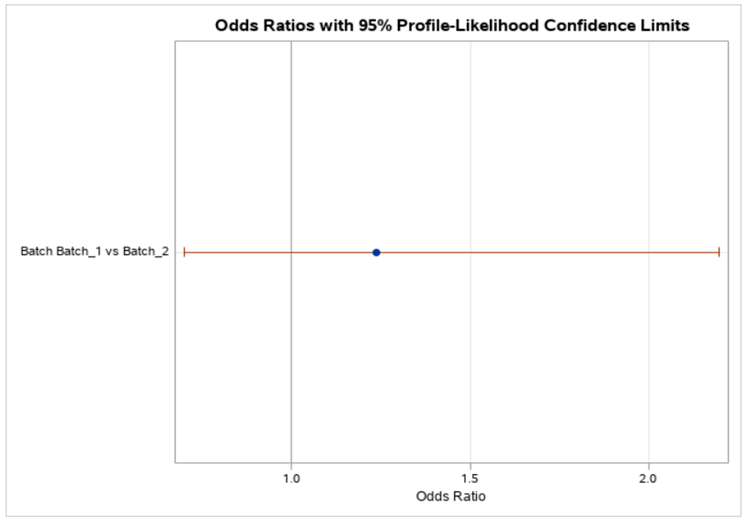


**Fig.6. Odds ratio, confidence interval for across the batch**


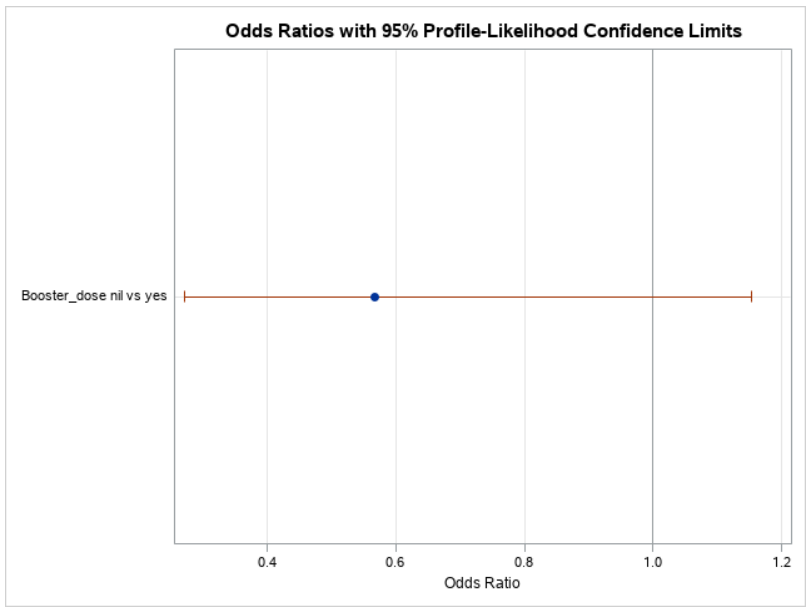


**Fig.7. Odds ratio, confidence interval for booster dose**


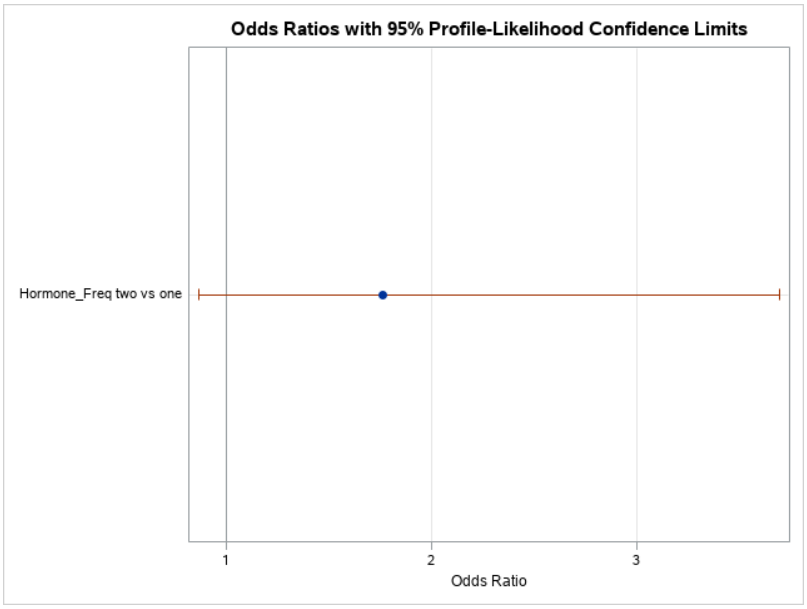


**Fig.8. Odds ratio, confidence interval for hormone frequency**


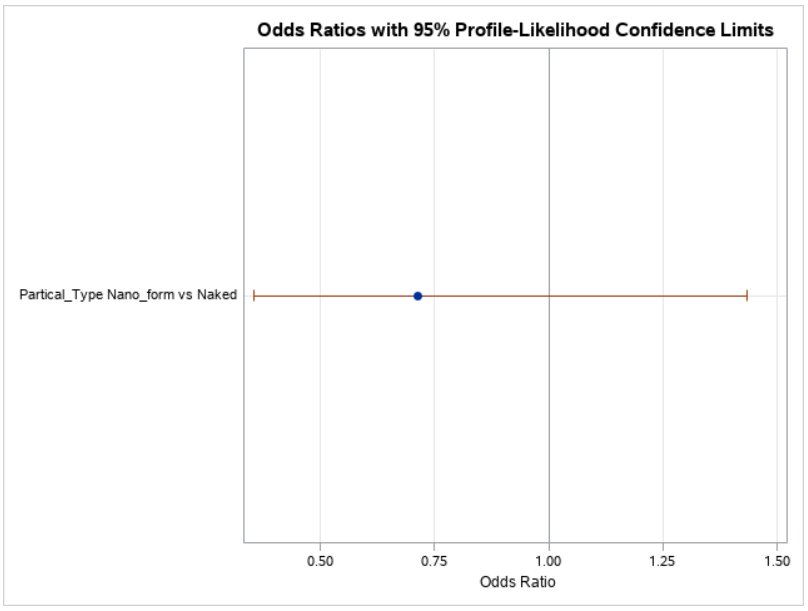


**Fig.9. Odds ratio, confidence interval for Particle-type**


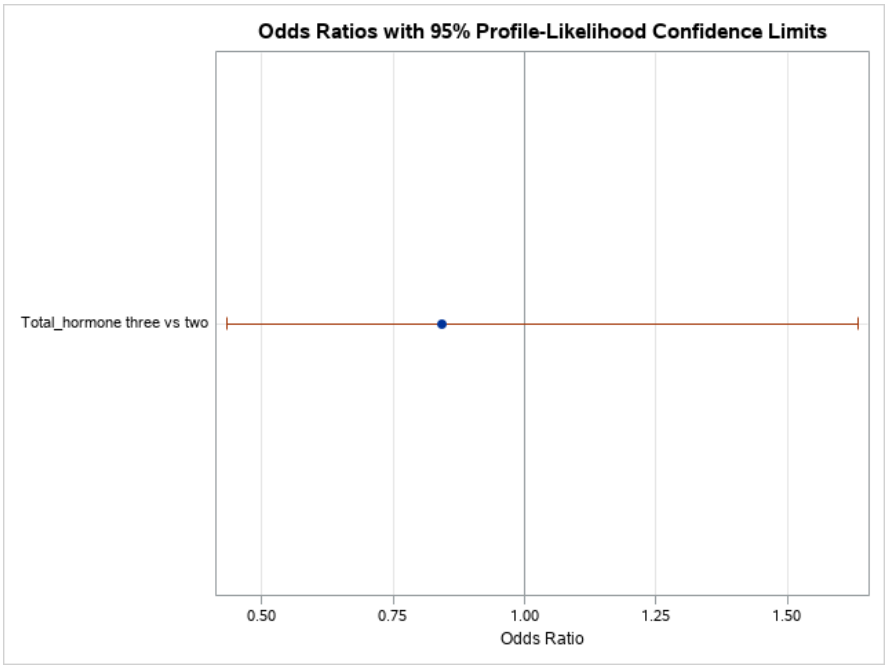


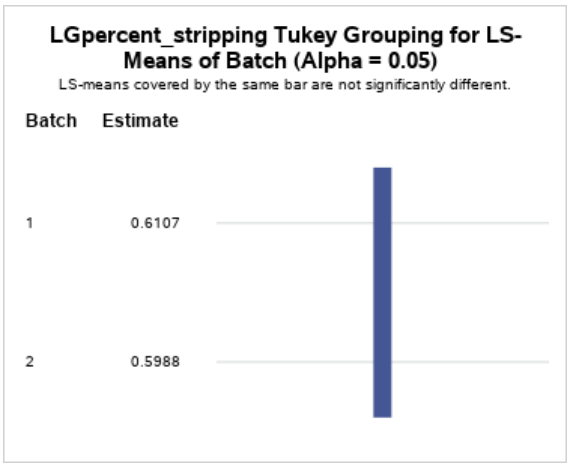
**Fig.10. Odds ratio, confidence interval for total hormone**

**Fig.11. Tukey-Kramer grouping for stripping percentage across batch**


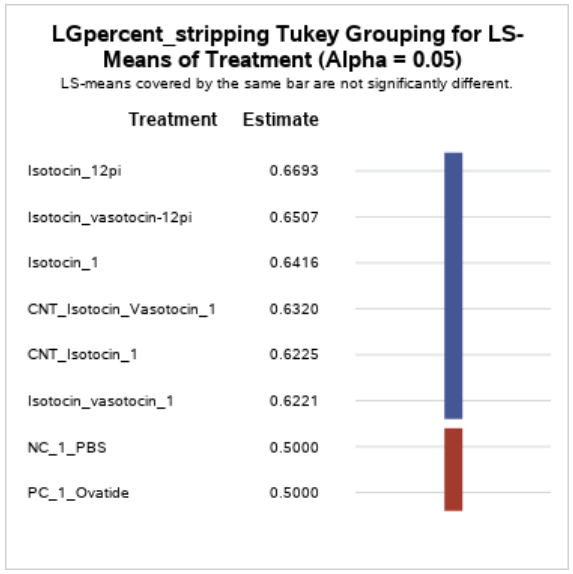


**
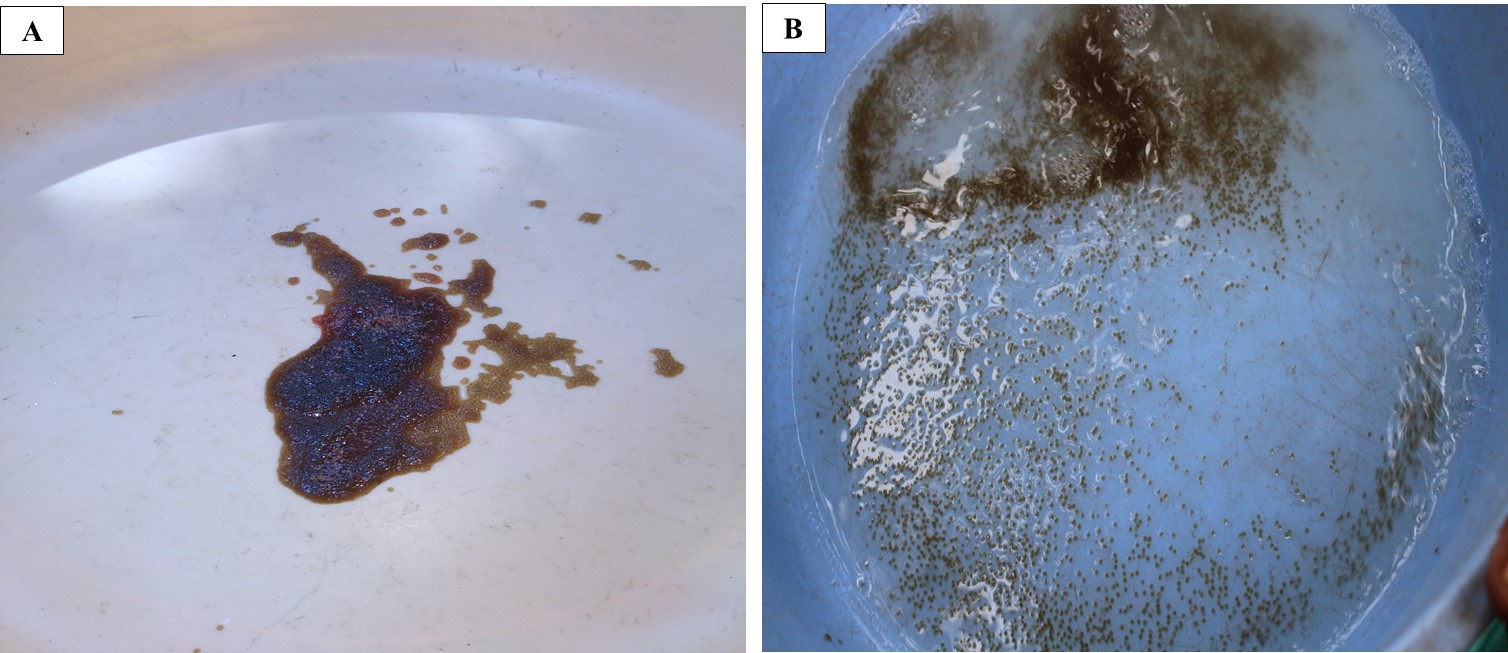
Fig.12. Tukey-Kramer grouping for stripping percentage across treatments**

**Fig.13. Quality eggs of *C. magur* (A) Mixing Eggs with collected Milt (B)**

**
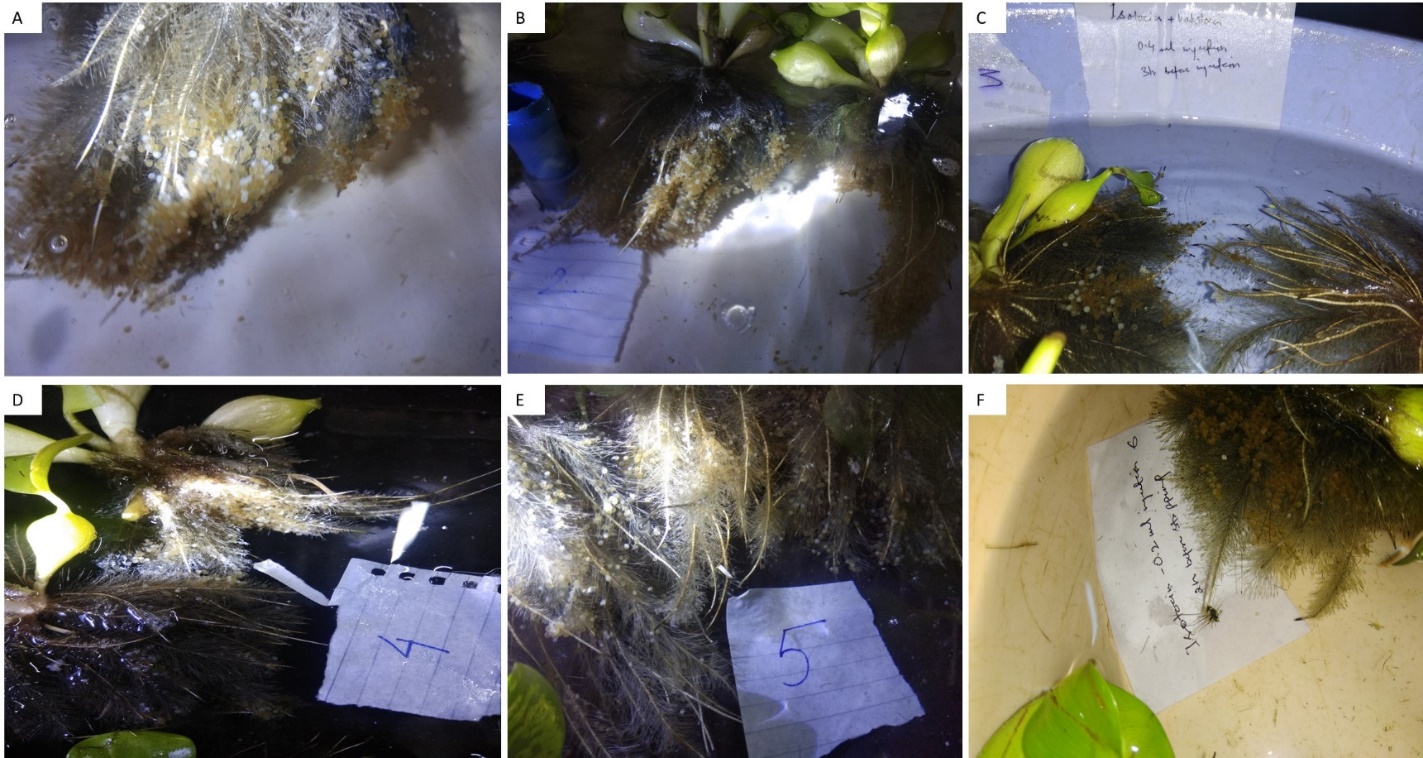
**

**Fig.14. Fertilized Eggs in *Eichhornia* Plant root and unfertilized eggs in whitish colour**


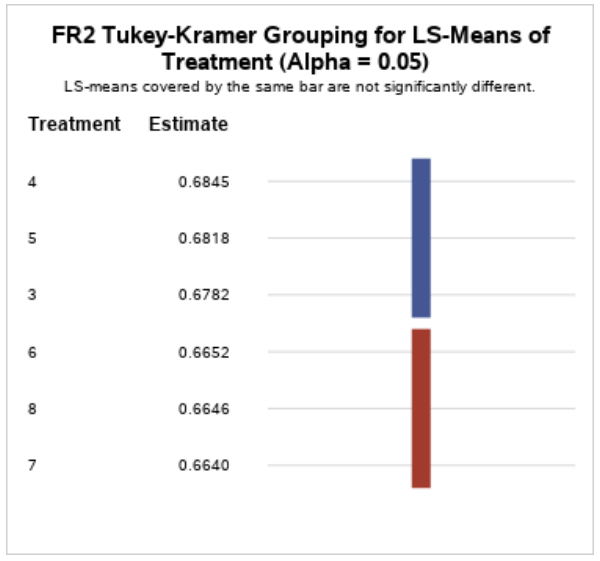


**Fig.15. Tukey-Kramer grouping for fertilization rate across treatments**


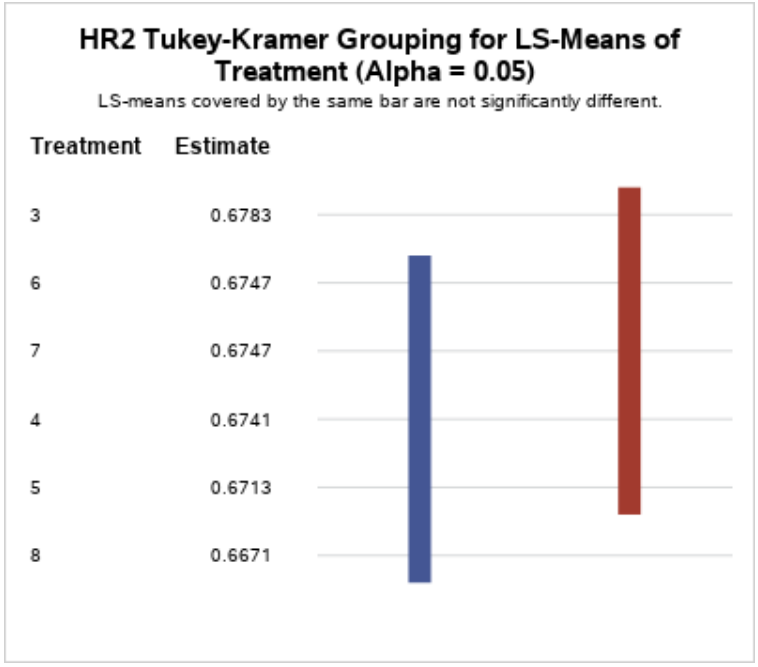


**Fig.16.Tukey-Kramer grouping for hatching rate across treatments**

**
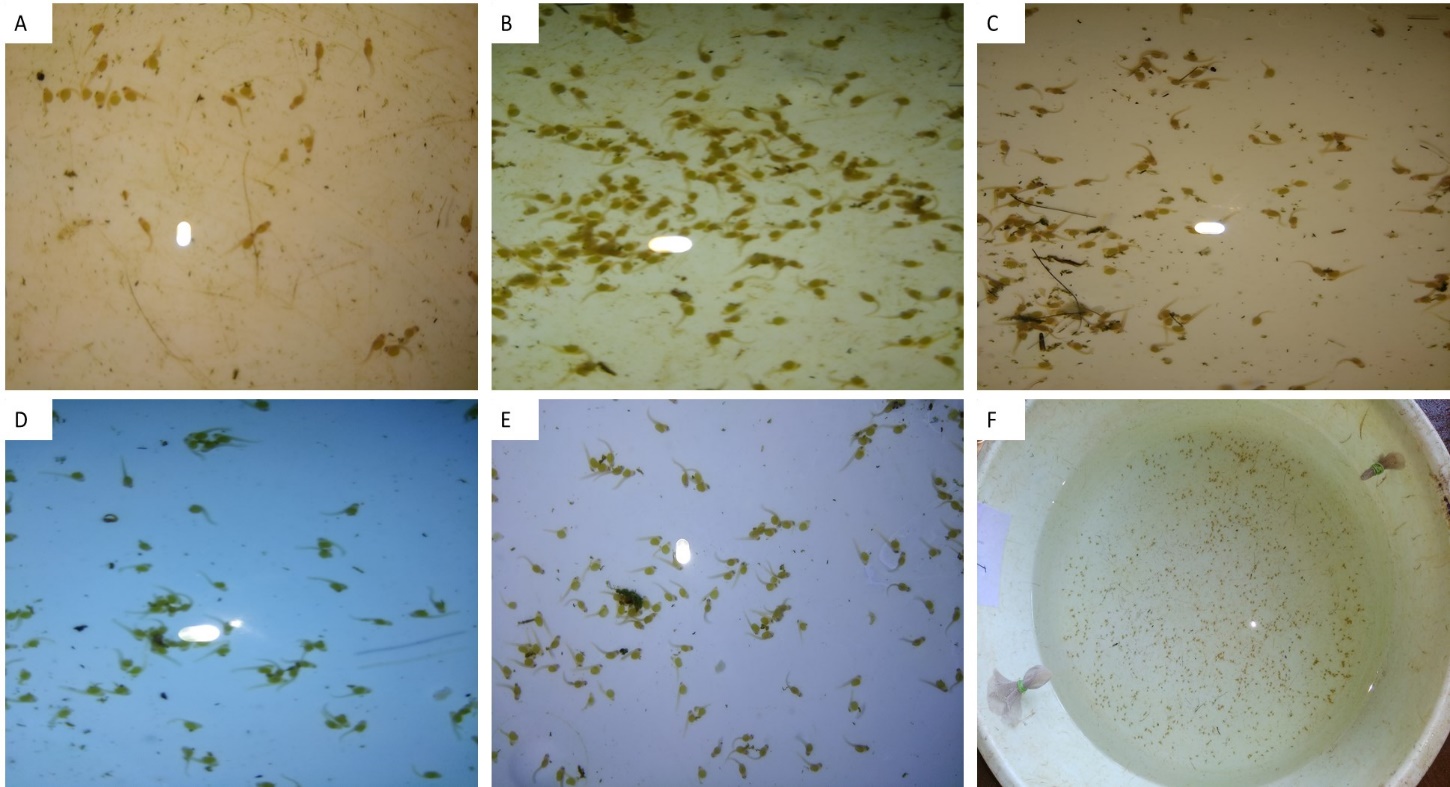
Fig.17. Hatchling of *C. magur***

**
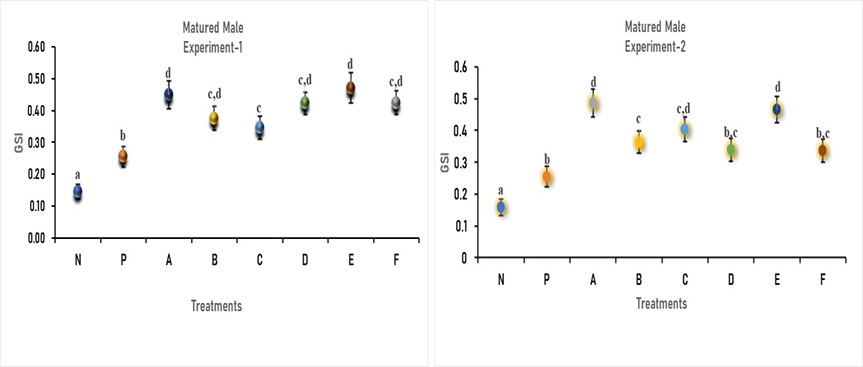
**

**Fig.18. GSI of male in different treatments**
